# Extra centrosomes delay DNA damage–driven tumorigenesis

**DOI:** 10.1126/sciadv.adk0564

**Published:** 2024-03-29

**Authors:** Vincent Z. Braun, Gerlinde Karbon, Fabian Schuler, Marina A. Schapfl, Johannes G. Weiss, Paul Y. Petermann, Diana C.J. Spierings, Andrea E. Tijhuis, Floris Foijer, Verena Labi, Andreas Villunger

**Affiliations:** ^1^ Institute for Developmental Immunology, Biocenter, Medical University of Innsbruck, Innsbruck, Austria.; ^2^ Department of Paediatrics I, Medical University of Innsbruck, Innsbruck, Austria.; ^3^ European Research Institute for the Biology of Ageing, University of Groningen, University Medical Center Groningen, Groningen, Netherlands.; ^4^ The CeMM Research Center for Molecular Medicine of the Austrian Academy of Sciences, Vienna, Austria.

## Abstract

Deregulated centrosome numbers are frequently found in human cancer and can promote malignancies in model organisms. Current research aims to clarify if extra centrosomes are cause or consequence of malignant transformation, and if their biogenesis can be targeted for therapy. Here, we show that oncogene-driven blood cancer is inert to genetic manipulation of centrosome numbers, whereas the formation of DNA damage–induced malignancies is delayed. We provide first evidence that this unexpected phenomenon is connected to extra centrosomes eliciting a pro-death signal engaging the apoptotic machinery. Apoptosis induction requires the PIDDosome multi-protein complex, as it can be abrogated by loss of any of its three components, *Caspase-2*, *Raidd/Cradd*, or *Pidd1*. BCL2 overexpression equally blocks cell death, documenting for the first time induction of mitochondrial apoptosis downstream of extra centrosomes. Our findings demonstrate context-dependent effects of centrosome amplification during transformation and ask to adjust current belief that extra centrosomes are intrinsically pro-tumorigenic.

## Teaser

Extra centrosomes can trigger PIDDosome-dependent mitochondrial apoptosis to limit malignant transformation elicited by DNA damage.

## INTRODUCTION

The centrosome is a membraneless organelle found in animal cells, which provides the structural basis for microtubule nucleation and related functions. Thus, centrosomes mediate chromosome segregation in mitosis, cell motility, organelle positioning and trafficking, cilia formation, as well as cell polarization (*1*). The centrosome consists of a mature mother centriole, carrying distal and subdistal appendage proteins, and a daughter centriole connected to its mother by the linker protein, cNAP1 (*2*). During S phase of the cell cycle, the genomic DNA and the centrosome are both duplicated. To secure chromosome separation in mitosis, two centrosomes are required to form the mitotic spindle, which allows microtubule nucleation from the two opposing spindle poles. After the mitotic spindle correctly captures and attaches sister chromatids, they are segregated toward the two opposing poles, followed by cytokinesis. Physical abscission results in two daughter cells, each containing a diploid set of chromosomes and one mature centrosome (*3*).

Polo-like kinase 4 (PLK4) plays a crucial role in the process of centriole duplication (*4*). Chemical inhibition of its function or genetic ablation leads to centriole loss that interferes with early development and normal cell proliferation (*5*). Conversely, overexpression of PLK4 leads to centriole amplification that can result in more than one mature centrosome per cell (*6*). Extra centrosomes can also result from cytokinesis failure or pathological cell fusion events, as well as during organogenesis to form naturally polyploid cells, e.g., in the liver, where hepatocytes proactively repress genes required for cytokinesis (*7*, *8*). Since extra centrosomes can cluster, a pseudo-bipolar spindle can allow successful cell division, a feature frequently enabling cancer cell growth (*9*). However, different numbers of centrioles at each spindle pole can lead to an altered microtubule nucleation equilibrium and cause faulty attachments of microtubules to kinetochores (*10*). This again can lead to mitotic delays and cause lagging chromosomes, or even rupture of chromosomes when trapped in the cytokinetic cleavage furrow (*11*). Even more catastrophic are situations where extra centrosomes fail to cluster and multipolar spindles arise. Subsequent multipolar mitoses can lead to general chromosomal segregation defects, resulting in aneuploidy, a phenomenon usually linked to proteotoxic stress and reduced cell fitness, autophagy, as well as cell death (*12*). These observations underlie the rationale to trial anticancer drugs that interfere with mitotic fidelity and cytokinesis, such as Aurora kinase inhibitors, as well as small molecules inhibiting members of the Polo-like kinase family members, including PLK1 and PLK4 (*13*). Moreover, γ-irradiation (IR) has been documented to cause centrosomal abnormalities that may become relevant in the context of cancer therapy, cancer evolution, and disease recurrence (*14*).

Despite this intrinsic fitness penalty, some cells manage to survive despite deregulated centrosome number that can promote genomic instability leading to aneuploidy and ultimately drive cancer formation (*15*). Consistently, extra centrosomes are frequently found in solid and hematological tumors and are often correlated with a poor prognosis (*7*, *16*). Common oncogenic drivers of blood cancer, like MYC (*17*) or BCR-ABL1, are linked to centrosome amplification in experimental models, and in the latter case, p210 BCR-ABL1 is reportedly a centrosome-associated protein in chronic myeloid leukemia cells (*18*, *19*).

Despite the clear link of extra centrosomes with cancer in humans, experimental models exploiting cell type–restricted overexpression of PLK4 revealed conflicting results about the ability of extra centrosomes to drive tumor formation. Conditional *Plk4* transgene expression in the skin or liver failed to promote spontaneous tumorigenesis (*20*). However, transient PLK4 overexpression enabled skin cancer development when p53 function was temporarily impaired in parallel (*21*). A direct role in cancer development has also been established in animal models exploiting conditional overexpression of PLK4 in all somatic tissues (*22*, *23*). Again, impaired p53 function appeared to enhance the cancer-promoting potential of extra centrosomes generated in these models (*23*, *24*). This simultaneously highlights the importance of mechanisms that restrict the growth of cells carrying extra centrosomes. One such cellular mechanism is monitoring the number of mature centrosomes present in a given cell. This is achieved by the PIDDosome, a multiprotein complex, consisting of p53-induced death domain protein 1 (PIDD1) and RIP-associated ICH1/CED3-homologous protein with death domain (RAIDD, also known as CRADD) that recruit the pro-enzymatic form of a protease, caspase-2, to promote cell cycle arrest (*25*).

*PIDD1*, initially identified as a p53 target gene and implicated along with caspase-2 in cell death induced by genotoxic DNA damage (*26*), localizes to the mother centriole in healthy cells via the distal appendix protein, ankyrin repeat domain containing 26 (ANKRD26) (*27*). Accumulation of an additional mature centriole, e.g., observed after cytokinesis failure, suffices to trigger PIDDosome formation. This leads to the auto-processing of caspase-2, a cysteine-directed and aspartate-specific endopeptidase. Caspase-2 cleaves and thereby inactivates the E3 ligase MDM2, which is the major inhibitor of p53 protein accumulation. As a consequence, PIDDosome activation leads to p53-dependent and p21-mediated cell cycle arrest in epithelial cancer cells accumulating extra centrosomes (*25*), as well as in primary hepatocytes during development and regeneration (*8*). It is unclear at present if the PIDDosome limits the growth of cells with extra centrosomes solely by promoting cell cycle arrest or if it is able to limit the transformation of such cells by other means.

Here, we investigate the impact of extra centrosomes induced by *Plk4* overexpression on oncogene- and DNA damage–driven tumorigenesis. While *Plk4* overexpression does not affect oncogene-driven blood cancer, lymphomagenesis and fibrosarcoma formation initiated by DNA damage are found delayed in the presence of a functional PIDDosome. This delay in transformation involves PIDDosome-initiated cell death in cells experiencing centrosome abnormalities in conjunction with DNA damage. Our findings demonstrate context-dependent effects of centrosome aberrations on cancer formation and identify extra centrosomes as triggers of mitochondrial apoptosis.

## RESULTS

### PLK4 overexpression does not affect oncogene-driven blood cancer

Lymphomas and leukemia are frequently driven by oncogene amplification or chromosomal translocations. Here, we tested the hypothesis that deregulation of the centrosome cycle, along with excessive proliferation, caused by overexpression of oncogenic MYC or aberrant expression of ABL tyrosine kinase, can accelerate lymphomagenesis or leukemia formation, respectively.

**Fig. 1.**
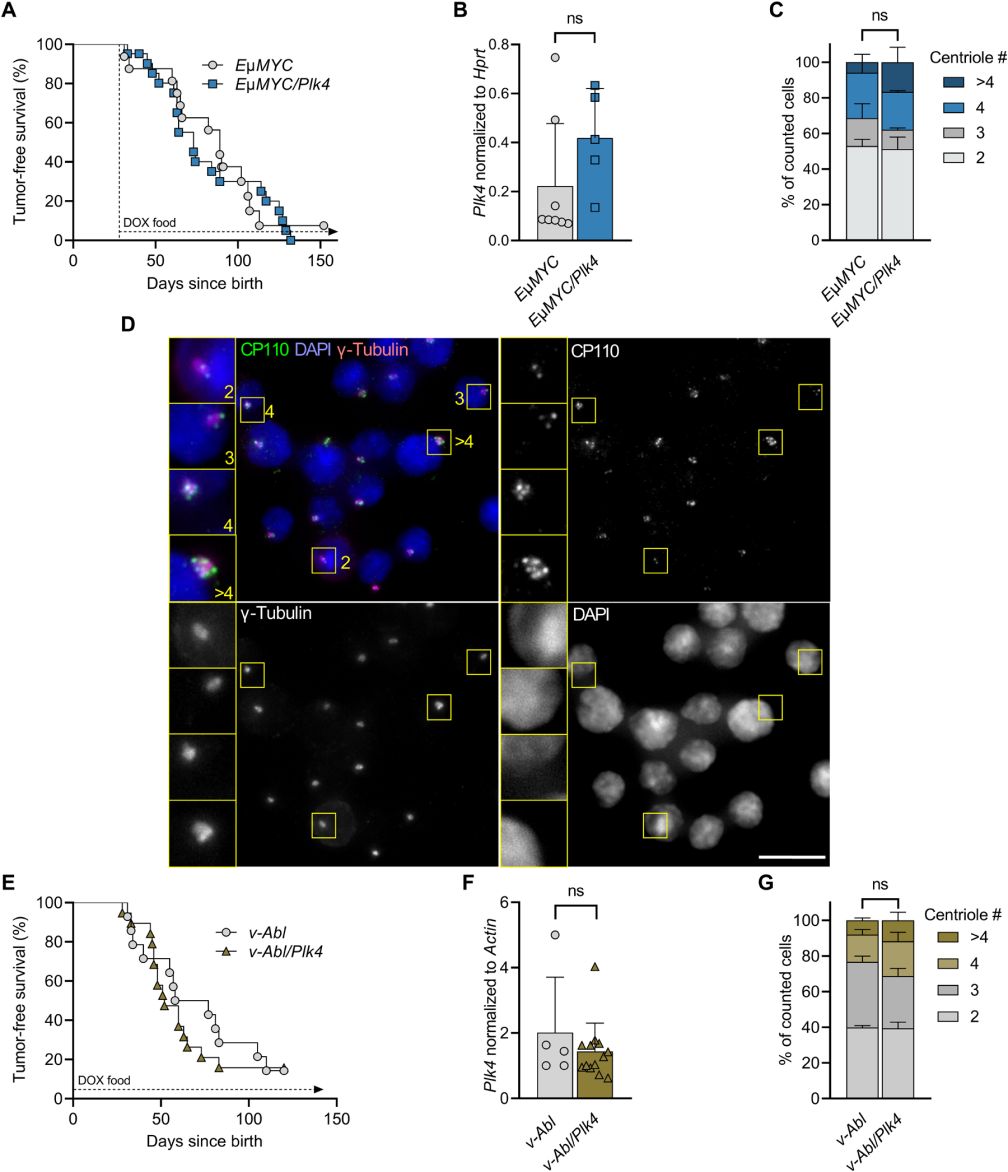
*E*μ*MYC*- and *v-Abl*–driven tumor onset is unaffected by PLK4 transgene expression. (**A**) Kaplan-Meier analysis of tumor-free survival of *E*μ*MYC* and *E*μ*MYC/Plk4* transgenic mice kept on doxycycline (DOX)–containing food after weaning (day 28) until disease onset. *E*μ*MYC* median survival 89 days (*n* = 15), *E*μ*MYC/Plk4* median survival 73 days (*n* = 18). Log-rank (Mantel-Cox) *P* = 0.8009 (χ^2^ = 0.06362), Breslow-Gehan-Wilcoxon *P* = 0.65 (χ^2^ = 0.1963). (**B**) *Plk4* mRNA expression assessed in tumors, which developed in *E*μ*MYC* (*n* = 8) or *E*μ*MYC/Plk4* mice (*n* = 5), normalized to *Hprt*. (**C**) Percentage of *E*μ*MYC* and *E*μ*MYC/Plk4* tumor cells presenting with 2, 3, 4, or >4 centrioles (*n* = 3 per genotype). (**D**) Example microscopy pictures of CP110 foci, marking centrioles in *E*μ*MYC* and *E*μ*MYC/Plk4* tumor cells (scale bar, 30 μm). (**E**) Kaplan-Meier analysis of tumor-free survival of *v-Abl*–infected *R26rtTA* (*n* = 13, median survival 67.5 days) and *R26rtTA/Plk4* (*n* = 14) transgenic mice (median survival 52 days). Pups received doxycycline before weaning via breastfeeding from their mothers that were kept on doxycycline. Log-rank (Mantel-Cox) *P* = 0.6097 (χ^2^ = 0.2607), Breslow-Gehan-Wilcoxon *P* = 0.5611 (χ^2^ = 0.3378). (**F**) *Plk4* mRNA expression in tumors that developed in *R26rtTA* (*n* = 5) and *R26rtTA/Plk4* (*n* = 9) mice after infection with *v-Abl*, normalized to *Actin*. (**G**) Percentage of *v-Abl*–driven tumor cells presenting with 2, 3, 4, or >4 centrioles isolated from *R26rtTA* (*n* = 2) and *R26rtTA/Plk4* (*n* = 3) mice. *Plk4* expression between genotypes was compared using the unpaired *t* test. Centriole counts were analyzed by Sidak’s multiple comparisons test. Data are shown as means ± SD. *P < 0.05, **P < 0.01, ***P < 0.005, ns = not significant.

First, we intercrossed *TET-Plk4* mice carrying the reverse tet-transactivator (*rtTA*) in the *Rosa26* locus (*R26rtTA*) with *E*μ*MYC* transgenic mice. These mice express the oncogene in early B cell progenitors and develop lymphomas within the first year of life (*28*). *R26rtTA E*μ*MYC* and *R26rtTA/TET-Plk4 E*μ*MYC* (referred to as *E*μ*MYC* and *E*μ*MYC/Plk4* mice in text and figures) were put on doxycycline-containing food after weaning and followed until disease onset. Tumor latency was comparable between *E*μ*MYC* and *E*μ*MYC/Plk4* mice (Fig. 1A). Similarly, tumor burden was found to be comparable between groups, and immunophenotyping did not reveal an obvious redistribution of pro/pre-B versus IgM^+^D^−^ immature B cell lymphomas (fig. S1, A and B), the two major lymphoma types arising in this model system (*28*).

Expression analysis further revealed that *Plk4* mRNA was not significantly increased in freshly isolated tumor cells from *E*μ*MYC/Plk4* mice (Fig. 1B). Consistently, immunofluorescence (IF) analysis for CP110 and γ-tubulin confirmed that the number of tumor cells with extra centrioles was not different between genotypes (Fig. 1, C and D). Together, this suggested impairment of *Plk4* transgene expression in *E*μ*MYC* transgenic B cell progenitors that ultimately give rise to disease.

Next, we exploited a mouse model for ABL kinase–driven pro/pre-B acute lymphoblastic leukemia (ALL), where neonatal mice are infected with *v-Abl* encoding retrovirus by subcutaneous injection straight after birth (*29*). Mothers were put on doxycycline-containing food, and expression of PLK4 was approximated by a surrogate doxycycline-responsive green fluorescent protein (GFP) reporter found to be active in up to 90% of CD19^+^ peripheral blood mononuclear cells in suckling pubs (fig. S1C). Cell lines established from tumor bearing mice, cultured ex vivo (fig. S1D), showed *Plk4* overexpression upon addition of doxycycline, indicating that the transgene locus was not systematically silenced. *R26rtTA* and *R26rtTA/TET-Plk4* mice infected with *v-Abl* (referred to as *v-Abl* and *v-Abl/Plk4* in text and figures) develop mainly CD43^+^ progenitor B cell leukemia (fig. S1E) (*29*)*.* Similar to our findings in *E*μ*MYC* transgenic mice, tumor latency did not differ between genotypes (Fig. 1E). Also, tumor cells did not show significantly increased *Plk4* mRNA levels straight after isolation from diseased animals, nor deregulated centriole counts, as monitored by microscopy (Fig. 1, F and G).

The transcription factor p53 is an established suppressor of disease in both model systems analyzed here (*30*–*33*), and loss of p53 allows proliferation of cells with amplified centrosomes (*34*). Hence, we evaluated the p53 status of a subset of tumors by Western blot analysis using p53 and p19ARF protein levels as a readout. p19ARF is activated in response to oncogenic stress to block MDM2 allowing p53 stabilization but repressed by p53 itself in a negative feedback loop (*30*, *35*). As such, high levels of p19ARF indicate abrogated p53 function, due to either mutation or deletion, while high p53 levels indicate inactivation by mutation, as neither MDM2 can be induced to degrade it nor p19ARF can be repressed. On the basis of this, we concluded that inactivation of p53 occurs in both disease models at comparable rates, with two of six *E*μ*MYC* and two of six *E*μ*MYC/Plk4* tumors showing clear signs of p53 pathway inactivation. *v-Abl* tumors also showed similar rates of pathway inactivation, with three of five *v-Abl* and three of six *v-Abl/Plk4* tumors showing high levels of p19ARF (fig. S1F).

Together, this suggests that *Plk4* overexpression is poorly tolerated in transgenic progenitor B cells experiencing oncogenic stress and may be rapidly selected against. As a result, frequencies of p53 pathway alterations remain unchanged in these model systems. Whether *Plk4* overexpression can speed up disease in conjunction with oncogenic stress on a *p53* haploinsufficient background even further remains to be tested in adequate model systems.

### Centriole amplification triggers apoptosis in *E*μ*MYC* transgenic progenitor B cells

**Fig. 2.**
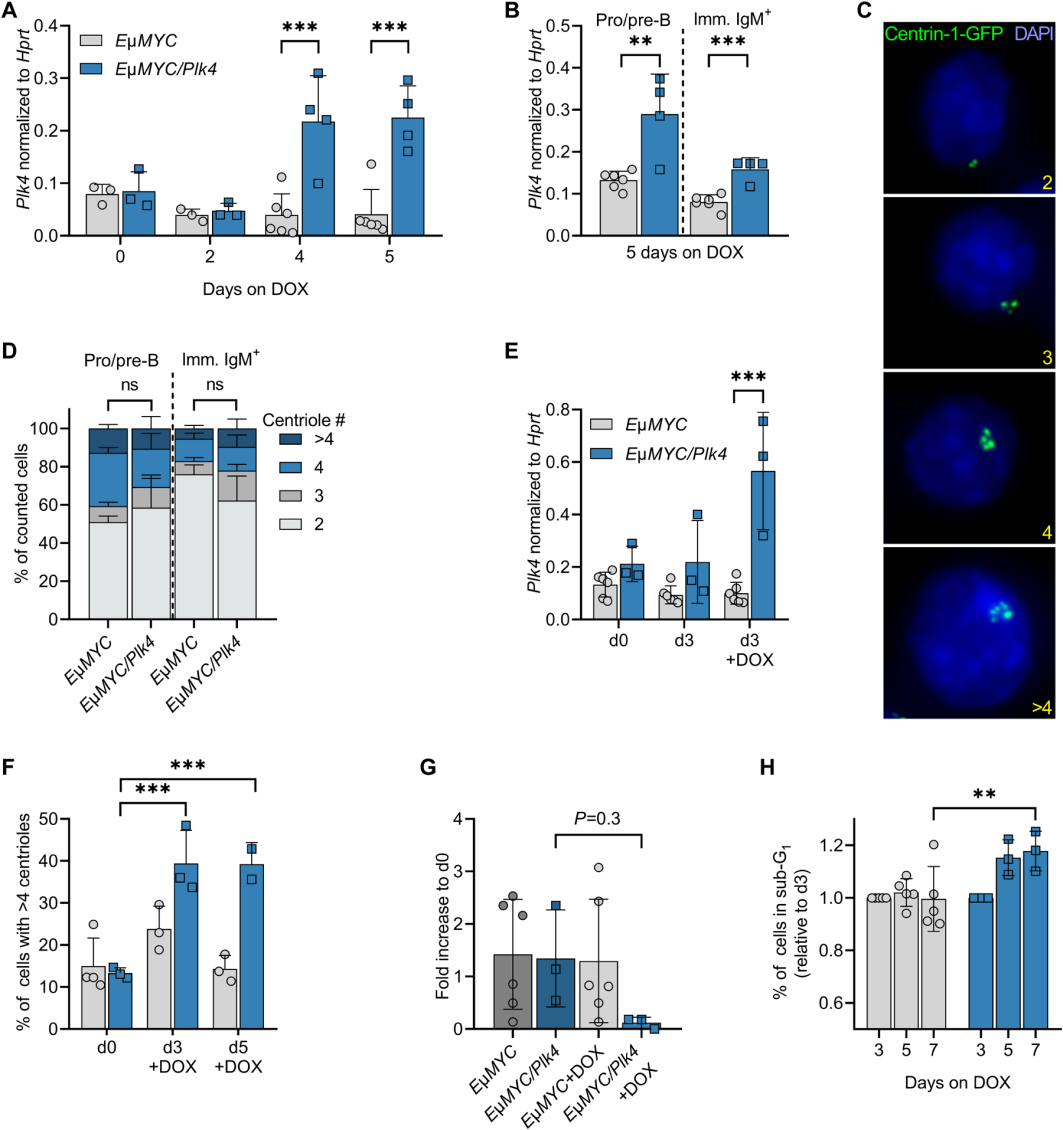
PLK4-induced centrosome amplification in B cells leads to cell death. (**A**) *Plk4* transgene expression in whole-blood samples of premalignant *E*μ*MYC* and *E*μ*MYC/Plk4* transgenic mice at the age of 5 weeks, kept on doxycycline for up to five additional days (*n* = 3 to 6). (**B**) *Plk4* expression in pro/pre-B and immature IgM^+^D^−^ B cells isolated by cell sorting from spleens of 5-week-old animals kept for 5 days on doxycycline. Expression was normalized to *Hprt* (*E*μ*MYC n* = 6; *E*μ*MYC/Plk4 n* = 5). (**C**) Example picture of Centrin-1-GFP foci marking centrioles (scale bar, 8 μm) in pro/pre-B and immature IgM^+^D^−^ B cells after isolation from spleens of 5-week-old *E*μ*MYC* and *E*μ*MYC/Plk4* transgenic mice, quantified in (D). (**D**) Percentage of *E*μ*MYC* (*n* = 3) and *E*μ*MYC/Plk4* (*n* = 4) pro/pre-B and immature IgM^+^ B cells presenting with 2, 3, 4, or >4 centrioles after 5 days on doxycycline-containing food. (**E**) *Plk4* transgene expression in pro-B cells isolated from *E*μ*MYC* (*n* = 6) and *E*μ*MYC/Plk4* mice (*n* = 3), cultured with or without doxycycline (1 μg/ml) for 3 days. Expression was normalized to *Hprt.* (**F**) Percentage of *E*μ*MYC* (*n* = 2) and *E*μ*MYC/Plk4* (*n* = 2) pro-B cells with >4 centrioles, cultured with or without doxycycline for 3 and 5 days. (**G**) Fold change of total cell number in pro-B cell cultures on day 7 normalized to day 0 (*n* = 3 to 6). (**H**) Percentage of sub-G_1_ cells in the respective pro-B cell cultures normalized to day 3 on days 5 and 7 (*E*μ*MYC n* = 5, *E*μ*MYC/Plk4 n* = 3). Data are shown as means ± SD and were statistically tested by Sidak’s multiple comparisons test. *P < 0.05, **P < 0.01, ***P < 0.005.

To test if PLK4 expression is detectable at least transiently, 28-day-old *E*μ*MYC/Plk4* mice were put on doxycycline right after weaning and analyzed 5 days later (day 33). Of note, samples of peripheral blood showed significantly increased levels of *Plk4* mRNA expression over time (Fig. 2A). In addition, cell-sorted progenitor B as well as IgM^+^ B cells isolated from the bone marrow also showed a discernible increase in transgene expression on day 5 (Fig. 2B).

To explore if the noted increase in mRNA eventually can translate into deregulated centriole numbers, EGFP-Centrin1 was crossed in as a centriole marker and cells were isolated from bone marrow by cell sorting and subjected to IF analyses (Fig. 2C). Despite increased mRNA levels, extra centrioles were not found in *E*μ*MYC* transgenic B cells inducing the *Plk4* transgene after exposure to doxycycline in vivo (Fig. 2D). In contrast to these observations, we noted that bone marrow–derived pro-B cells cultured in vitro showed *Plk4* mRNA overexpression (Fig. 2E), going hand in hand with an increase in extra centrioles (Fig. 2F). On top of this, we observed a clear drop in cell number (Fig. 2G) and a significant increase in the fraction of sub-G_1_ cells, indicative of increased apoptosis in *Plk4*-overexpressing cells (Fig. 2H and fig. S2A). This indicates that centrosome-amplifying double-transgenic progenitor B cells die at increased rates and are likely readily cleared in vivo, whereas this phenomenon can still be detected in vitro in the absence of efferocytosis.

We conclude that oncogene-driven proliferation stress, in combination with centriole amplification, creates a cell death–inducing signal, leading to the rapid loss of such cells in situ. Our findings suggest that, in the context of oncogenic stress, extra centrioles fail to initiate cancer. However, they may still contribute to tumor evolution. Hence, we wondered if the karyotype in these tumors may differ in their level of aneuploidy. Whole-genome sequencing (WGS) of tumor cells in mini-bulks (30 cells per tumor) showed a trend toward enhanced karyotypic alterations on the *E*μ*MYC/Plk4* transgenic background, but statistical significance was not reached (fig. S2, B and C).

Together, our findings indicate that *Plk4* overexpression–induced centrosome amplification promotes cell death in progenitor B cells, at least in the presence of oncogenic MYC or ABL, curtailing potential pro-tumorigenic effects.

### Transient PLK4 overexpression delays DNA damage–driven transformation

Of note, transient *Plk4* overexpression has been shown to accelerate spontaneous thymic lymphomagenesis when *p53* function was simultaneously compromised (*22*, *23*). Hence, we decided to investigate oncogenicity in T cells, driven by DNA damage, and asked if extra centrioles would synergize with IR to speed up disease in this model. Repeated low-dose IR damage (4 × 1.75 Gy in weekly intervals) drives the formation of thymic lymphomas in mice, which is suppressed by p53 (*36*). In this model, transformation depends on repeated cycles of compensatory proliferation of hematopoietic stem and progenitor cells (HSPCs) in response to systemic IR-induced myelosuppression (*37*, *38*).

**Fig. 3.**
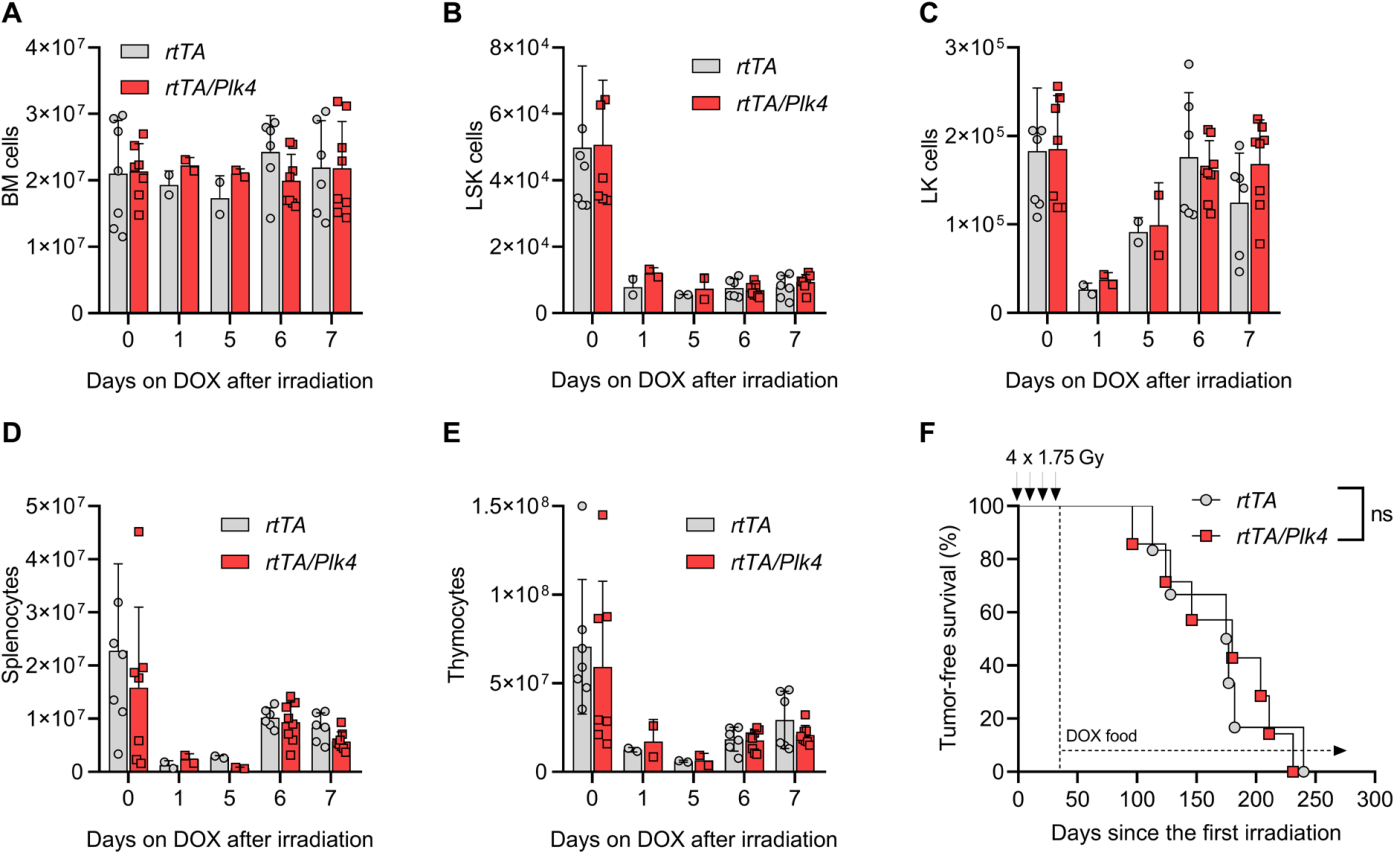
Chronic PLK4 transgene activation does not affect hematopoietic recovery or transformation after IR. *rtTA* and *rtTA/Plk4* mice, 4 weeks of age, were exposed to a single dose of 1.75-Gy IR and kept on doxycycline for up to 7 days. (**A**) Bone marrow (BM) cellularity of two femora/animal was defined by cell counting in a hemocytometer. (**B**) Flow cytometry–based assessment of cellularity of the LSK cell fraction in (A). (**C**) Cellularity of the LK cell fraction in (A). (**D**) Cell numbers of spleen and (**E**) thymus were assessed at the indicated times in *rtTA* and *rtTA/Plk4* transgenic mice kept on doxycycline-containing chow for up to 5 days after 1.75-Gy IR. (**F**) Kaplan-Meier analysis of tumor-free survival of *rtTA* and *rtTA/Plk4* transgenic mice fed with doxycycline-containing chow after the last IR cycle (4 × 1.75 Gy). *rtTA*, median survival 176 days (*n* = 6); *rtTA/Plk4*, median survival 180 days (*n* = 7). Log-rank (Mantel-Cox) *P* = 0.9501 (χ^2^ = 0.003911), Breslow-Gehan-Wilcoxon *P* = 0.8878 (χ^2^ = 0.01990). Cell count data are shown as means ± SD and tested by Sidak’s multiple comparisons test. *P < 0.05, **P < 0.01, ***P < 0.005.

First, we monitored the in vivo recovery potential of different leukocyte populations after a single dose of IR. We did not recognize an impact of *Plk4* transgene activation on the total number of bone marrow cells, including the number of Lin^−^Sca1^+^c-Kit^+^ (LSK) cells, a population enriched in HSPCs, or lineage-committed Lin^−^c-Kit^+^ (LK) cells, isolated from animals subjected to IR and fed with doxycycline-containing chow for the following 7 days (Fig. 3, A to C, and fig. S3A). Similarly, the number of thymocytes and mature splenocytes was comparable between genotypes 1 week after IR in the absence or presence of doxycycline (Fig. 3, D and E). Moreover, IR-induced thymocyte apoptosis ex vivo was also unaffected by increases in *Plk4* mRNA expression (fig. S3, B and C). This led us to conclude that tissue damage and depletion after IR damage, as well as cell death susceptibility, are comparable between genotypes.

Next, we exposed *R26rtTA* or *R26rtTA/TET-Plk4* transgenic littermates (referred to as *rtTA* or *rtTA/Plk4* in the figures) to fractionated IR of 4 × 1.75 Gy, starting at the age of 4 weeks. Mice were put continuously on doxycycline diet 1 week after the last round of irradiation. Following these animals until disease onset revealed that continuous application of doxycycline had no impact on IR-induced tumorigenesis (Fig. 3F). Also, tumor burden was found to be comparable between groups and immunophenotyping did not reveal differences in the frequencies of CD4^+^, CD8^+^, or mixed thymic lymphomas (fig. S3, E to G). Similar to tumors isolated from *v-Abl/Plk4 and E*μ*MYC/Plk4* transgenic mice, levels of *Plk4* mRNA were not significantly elevated in freshly isolated tumors (Fig. 1, B and F, and fig. S3D), suggesting transgene silencing. However, primary thymocyte cultures of adult mice responded with an increase in *Plk4* mRNA after doxycycline treatment (fig. S3B). Together, this suggests that lack of increased transgene expression in vivo may be due to loss of progenitor cells or thymocytes expressing high levels of *Plk4* mRNA. We hypothesized that this may potentially be due to cell death, rather than transgene silencing.

**Fig. 4.**
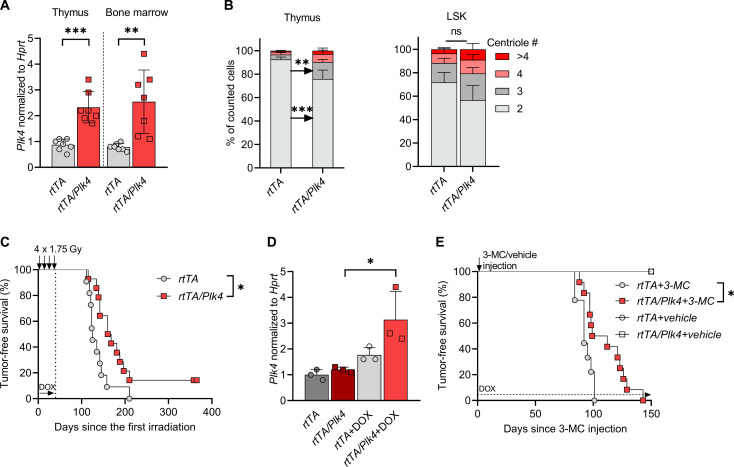
PLK4 overexpression can delay DNA damage–induced malignancies. (**A**) *Plk4* mRNA expression normalized to *Hprt* levels in thymus and total bone marrow isolated from *rtTA* (*n* = 7) and *rtTA/Plk4* (*n* = 7) transgenic mice fed with doxycycline-containing diet for 5 days. (**B**) Percentage of thymocytes or LSK cells presenting with 2, 3, 4, or >4 centrioles. Cells were isolated from *rtTA* (*n* = 2) and *rtTA/Plk4* (*n* = 2) transgenic mice fed with doxycycline-containing diet for 5 days. A minimum of 160 cells were counted per condition. (**C**) Kaplan-Meier analysis of tumor-free survival of mice kept on doxycycline during the weekly cycles of IR (4 × 1.75 Gy). *rtTA*, median survival 125 days (*n* = 11); *rtTA/Plk4*, median survival 164 days (*n* = 12). Log-rank (Mantel-Cox) *P* = 0.0159 (χ^2^ = 5.817), Breslow-Gehan-Wilcoxon *P* = 0.0099 (χ^2^ = 6.658). (**D**) *Plk4* mRNA expression in tumor cell lines established from diseased *rtTA* (*n* = 3) and *rtTA/Plk4* transgenic mice (*n* = 3) shown in (C), treated with doxycycline for 48 hours. (**E**) Kaplan-Meier analysis of tumor-free survival of *rtTA* (*n* = 9) and *rtTA/Plk4* (*n* = 12) transgenic mice injected with either 3-MC or vehicle. All animals were kept continuously on doxycycline-containing food. Median latency 92 versus 105.5 days. Log-rank (Mantel-Cox) *P* = 0.0130 (χ^2^ = 6.176), Breslow-Gehan-Wilcoxon *P* = 0.0205 (χ^2^ = 5.372). Tukey’s multiple comparisons test was used to compare *Plk4* mRNA data. Centriole quantifications data were tested by Sidak’s multiple comparisons test. *P < 0.05, **P < 0.01, ***P < 0.005.

Given the findings above, we reasoned that the selective pressure might change when *Plk4* is overexpressed transiently, during phases of high rates of compensatory proliferation between IR cycles. Therefore, we altered our strategy by providing doxycycline only during the recovery phases between IR cycles. Transgene expression in disease-relevant tissues was confirmed by mRNA isolation from thymus and bone marrow of mice kept on doxycycline-containing chow for 5 days, after the first dose of IR. We were able to confirm an increase in *Plk4* mRNA in both tissues (Fig. 4A). Of note, assessing centriole number in thymocytes and LSK cells isolated from mice that also harbored the EGFP-Centrin1 reporter revealed an increase in extra centrioles, although statistical significance was only achieved in the thymus (Fig. 4B). Regardless, we concluded that transient transgene expression and centrosome amplification can be achieved in these tissues and hence decided to monitor animals until disease onset.

Housing mice on standard chow after the last IR cycle revealed that tumor latency, against our expectations, was significantly delayed (Fig. 4C). Moreover, ex vivo cultured tumor cells established from these animals were still proficient in expressing the *Plk4* transgene upon doxycycline addition (Fig. 4D). To scrutinize this observation in a different p53-dependent tumor model system, we induced fibrosarcomas by intramuscular injection of the DNA-damaging agent 3-methylcholantrene (3-MC), a potent mutagen causing mostly G to T transversions (*39*). Animals were placed simultaneously on doxycycline-containing chow until tumors arose. Tumor latency was again significantly delayed in mice carrying the *Plk4* transgene (Fig. 4E). This suggests that the presence of extra centrosomes in conjunction with DNA damage can delay the rise of malignant clones. We reasoned that this phenomenon is linked to either cell cycle arrest and improved subsequent DNA repair, more effective removal of premalignant clones by the induction of cell death, or a combination of both.

### PLK4 overexpression limits HSPC growth and survival

**Fig. 5.**
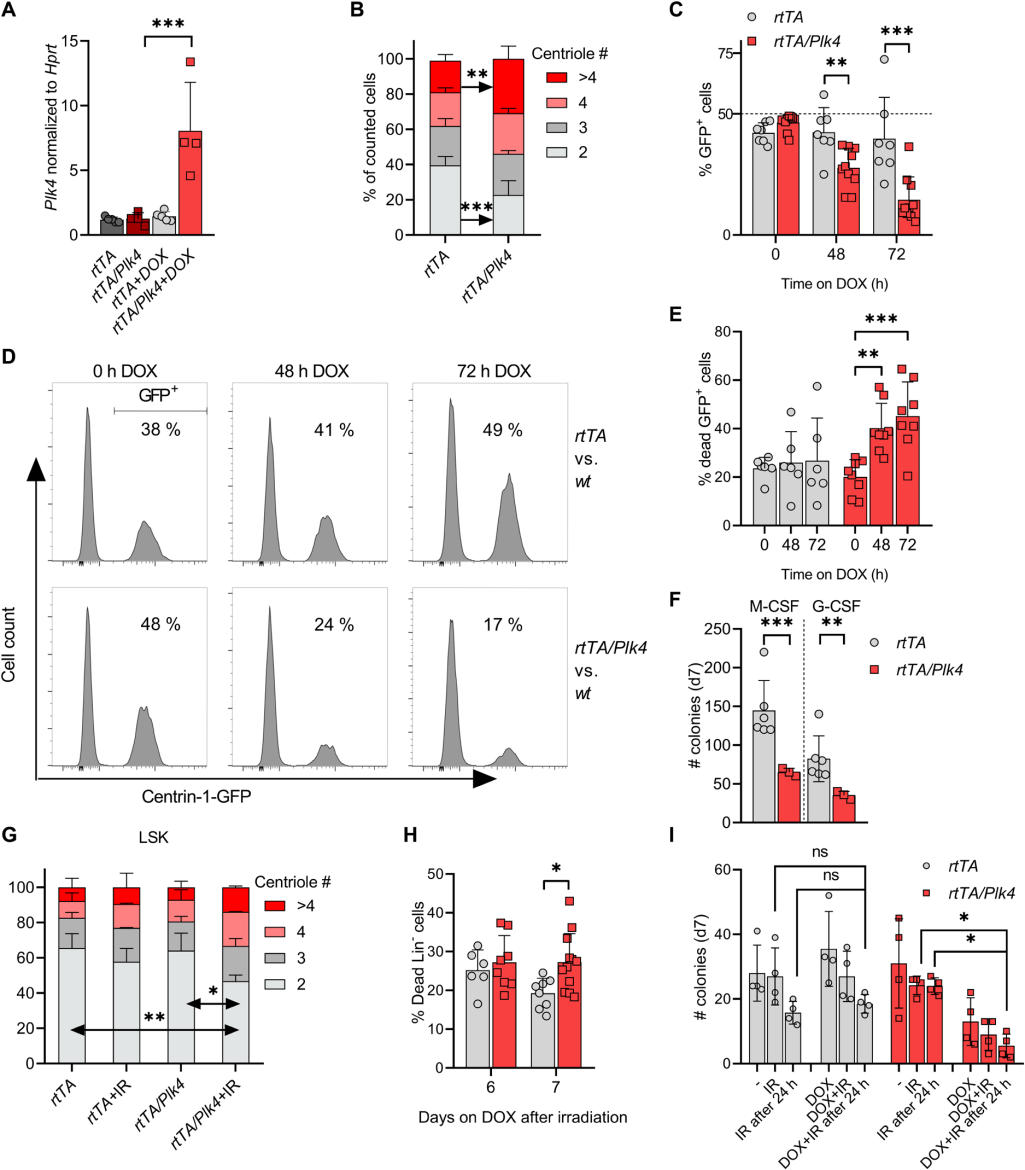
PLK4 overexpression limits the clonogenic potential of HSPCs. (**A**) *Plk4* mRNA in MPP cells was assessed after 48 hours of culture ± doxycycline and normalized to *Hprt* (*rtTA*, *n* = 5; *rtTA/Plk4*, *n* = 4). (**B**) MPPs from *rtTA* (*n* = 5) and *rtTA/Plk4* (*n* = 4) mice were cultured ± doxycycline for 48 hours to quantify centriole numbers. (**C**) Quantification of SMP competition assays, *rtTA* (two biological replicates, three technical replicates); *rtTA/Plk4* (five biological replicates, two technical replicates). (**D**) Representative histograms of cell competition assays using SMPs from *rtTA* mice, mixed 1:1 with SMPs from *rtTA* or *rtTA/Plk4 *mice expressing also EGFP-Centrin1, quantified in (C). (**E**) Quantification of cell death observed in (C). *rtTA* (two biological replicates, three technical replicates), *rtTA/Plk4* (four biological replicates, two technical replicates). (**F**) MethoCult assays using fetal livers from E14.5 embryos of *rtTA* (*n* = 6) and *rtTA/Plk4* mice (*n* = 3), cultured in the presence of doxycycline. (**G**) Percentage of LSK cells presenting with 2, 3, 4, or >4 centrioles. Cells were isolated from *rtTA* (*n* = 2) or *rtTA/Plk4* (*n* = 4) mice, fed with doxycycline for 5 days after 1.75-Gy IR. At least 160 cells were counted per condition. (**H**) *rtTA* and *rtTA/Plk4* mice 4 weeks of age were exposed to 1.75 Gy of IR and kept on doxycycline until analysis. Lin-negative cells from bone marrow were analyzed by forward/sideward-scatter separation to estimate viability. (**I**) M-CSF–induced colony formation using total bone marrow from 6-week-old *rtTA* (*n* = 4) and *rtTA/Plk4* mice (*n* = 4). Bone marrow was irradiated with 0.5 Gy before seeding or 24 hours after seeding. Doxycycline was applied immediately in both settings. Data are shown as means ± SD. qRT-PCR, fetal-liver MethoCult, and competition assay data were statistically tested by Tukey’s multiple comparisons test. BM-MethoCult and centriole quantification data were tested by Sidak’s multiple comparisons test. *P < 0.05, **P < 0.01, ***P < 0.005.

To evaluate the impact of PLK4 overexpression on HSPC fitness, we made use of FLT3-dependent multi-potent progenitors (MPPs) and stem cell factor (SCF)–dependent myeloid progenitors (SMPs) generated from *rtTA* and *rtTA/Plk4* bone marrow by transduction with the homeobox gene *HoxB8* (*40*, *41*). Culturing *rtTA/Plk4*-derived MPP in the presence of doxycycline allowed detection of increased *Plk4* mRNA expression alongside increased centriole numbers when compared to *rtTA* controls (Fig. 5, A and B). Next, we monitored the growth of SMP upon *Plk4* induction in cell competition assays. We observed that *rtTA/Plk4*-derived SMP, carrying EGFP-Centrin1 as a marker, were efficiently outcompeted by their wild-type counterparts when cultured in the presence of doxycycline (Fig. 5, C and D). This phenomenon coincided with increased cell death rates, arguing against a strong cell cycle arrest in SMP cells overexpressing *Plk4* (Fig. 5E and fig. S4A).

To corroborate our findings further, we performed colony formation assays in methylcellulose, using E14.5 fetal livers from *rtTA* and *rtTA/Plk4* and added cytokines, allowing formation of macrophage [macrophage colony-stimulating factor (M-CSF)] or granulocyte [granulocyte colony-stimulating factor (G-CSF)] colonies. Colony numbers were significantly reduced in the presence of doxycycline when analyzing fetal livers from *rtTA/Plk4* transgenic embryos (Fig. 5F), in line with an impaired clonogenic potential upon *Plk4* overexpression. Together, this suggests that HSPCs become primed to die upon *Plk4* induction*,* a situation potentially exacerbated by DNA damage. Of note, centriole amplification has been observed in G_2_-arrested cells after exposure to IR (*14*), raising the possibility that PLK4 overexpression and IR may act in an additive manner. To investigate this, we exposed mice to a single dose of IR and administered doxycycline for 5 days to induce transgene expression. Control animals received only doxycycline via chow. LSK cells were isolated by cell sorting, and centriole counts were analyzed by IF. The combination of IR plus doxycycline induced highest centriole counts in situ (Fig. 5G). This observation also correlated with increased rates of cell death within the Lin-negative pool of bone marrow cells on day 7 (Fig. 5H and fig. S4B). Moreover, low-dose irradiation in combination with *Plk4* overexpression also showed a clear trend to reduce the colony formation capacity of HSPCs in methylcellulose even further, when compared to doxycycline treatment alone (Fig. 5I).

Together, these results indicate that *Plk4* overexpression reduces the fitness of HSPCs, likely by induction of cell death, and that this effect appears exacerbated after IR damage. How cell death is initiated under these conditions remained uncertain, but given the proposed role of PIDD1 as a sensor of extra centrosomes and caspase-2 as a cell death initiator, we hypothesized that the PIDDosome multi-protein complex might contribute here.

### PLK4-driven PIDDosome activation promotes mitochondrial apoptosis

**Fig. 6.**
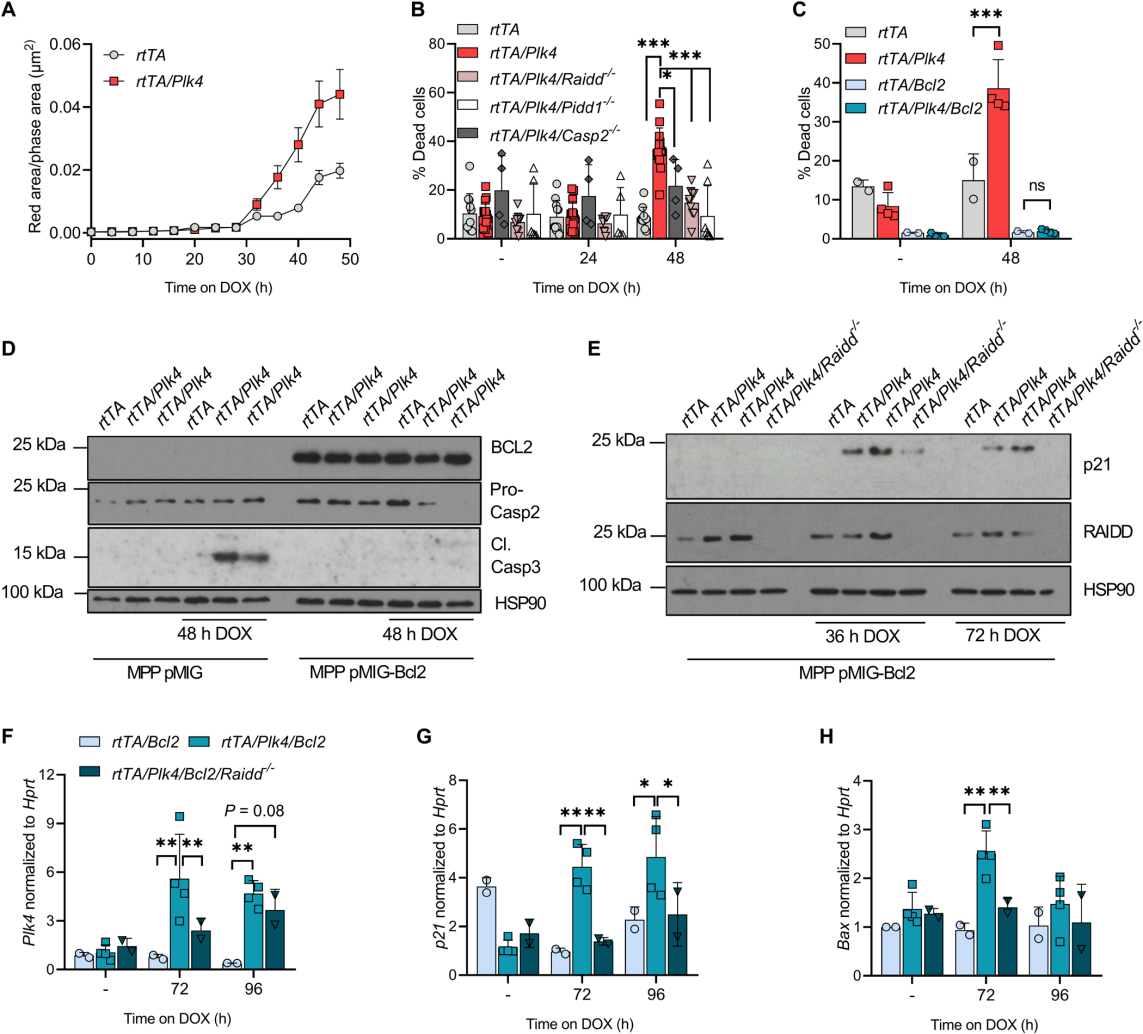
Centrosome amplification leads to PIDDosome-dependent cell death. (**A**) Time-dependent caspase-3 substrate processing in multipotent hematopoietic progenitors (MPP) after doxycycline treatment was measured over time by IncuCyte analysis (red fluorescence area/phase area in μm^2^). *rtTA* (five biological replicates), *rtTA/Plk4* (four biological replicates). (**B**) Quantification of DAPI and annexin V–positive MPP cells after 24 or 48 hours of doxycycline exposure. *rtTA* (two biological replicates), *rtTA/Plk4* (four biological replicates), *rtTA/Plk4/Casp2^−/−^* (four biological replicates), *rtTA/Plk4/Raidd^−/−^* (two biological replicates), *rtTA/Plk4/Pidd1^−/−^* (two biological replicates). Two to five technical replicates were performed. (**C**) Flow cytometric evaluation of DAPI and annexin V staining of MPP of the indicated genotypes after 48 hours of doxycycline exposure. pMIG retroviral expression vectors were used to transduce MPPs of the indicated genotypes with human BCL2. *rtTA* (*n* = 1), *rtTA/Plk4* (two biological replicates); *rtTA/Bcl2* (*n* = 1), *rtTA/Plk4/Bcl2* (two biological replicates). Two technical replicates were performed. (**D**) Western blot analysis of pMIG and pMIG BCL2-transduced MPP kept 48 hours on doxycycline. Two technical replicates were performed. (**E**) Western blot analysis of MPP protein extracts isolated from MPPs of the indicated genotypes 36 or 72 hours after addition of doxycycline. Two technical replicates were performed. (**F**) qRT-PCR analysis of *Plk4*, (**G**) *p21*, and (**H**) *Bax* mRNA in BCL2-overexpressing MPPs of the indicated cell lines in (E), kept for 72 or 96 hours on doxycycline. Two technical replicates were performed. Data are shown as means ± SD and tested by Sidak’s multiple comparisons test. *P < 0.05, **P < 0.01, ***P < 0.005.

To discriminate reduced proliferation from cell death, we monitored FLT3-dependent MPP over time by live cell imaging. This analysis revealed that *Plk4*-overexpressing cells showed higher caspase-3 reporter activation, starting after about 30 hours of transgene induction (Fig. 6A and fig. S4C), a time span expected to allow transgene expression, centrosome amplification, and mitotic traverse, needed for PIDDosome activation (*25*). Caspase-3 reporter activity seen in control cells was clearly less pronounced, and the delayed increase likely reflects spontaneous cell death in expanding cultures. Parallel flow cytometric analysis coincided with propidium iodide (PI) uptake and binding of annexin V, suggesting induction of apoptotic cell death (fig. S4D). To test if this cell death was PIDDosome dependent, we created MPPs lacking individual PIDDosome components and monitored cell survival in the presence of doxycycline. Loss of *Caspase-2*, *Raidd*, or *Pidd1* could reduce the number of dead cells in the presence of *Plk4* mRNA overexpression (Fig. 6B). This led us to postulate that *Plk4* overexpression can engage the PIDDosome in response to extra centrosomes for the initiation of cell death. To confirm p53 activation downstream of the PIDDosome, before induction of apoptosis, we also monitored mRNA levels of the p53 target genes, *p21* and *Bax*. Their expression was not increased after activation of the *Plk4* transgene with time, questioning the induction of the expected p53 response. However, we reasoned that the p53 signal may be masked by the induction of cell death noted in culture (Fig. 6, A and D, and fig. S4, C and D). Hence, the same experiment was repeated with an MPP variant overexpressing a BCL2 transgene that blocks mitochondrial apoptosis. While loss of PIDDosome function reduced cell death to some degree, BCL2 overexpression completely abrogated apoptosis, including the one spontaneously occurring in tissue culture, unaffected by PIDDosome deficiency (Fig. 6C). These findings were confirmed also on protein level, where cells overexpressing *Plk4* showed caspase-3 activation, while loss of the pro-form of caspase-2, a sign of its activation, was best noted in the context of BCL2 overexpression (Fig. 6D). Consistent with our idea, accumulation of p21 protein was best detectable on a BCL2 transgenic background (Fig. 6E). Moreover, *p21* and *Bax* mRNA induction in response to *Plk4* transgene activation were initially undetectable (fig. S4, E and F) unless cell death was inhibited by BCL2. Consistent with PIDDosome activation downstream of PLK4, this transcriptional response was dependent on the simultaneous presence of RAIDD (Fig. 6, F to H). We conclude that the p53 pathway can be engaged in MPPs, but may not be critical to drive mitochondrial apoptosis.

To gain support for our working model that extra centrosomes promote cell death downstream of *Plk4* overexpression, we considered alternative means to induce centrosome accumulation. Hence, we decided to induce cytokinesis failure by treating HoxB8 progenitors of different genetic backgrounds with dihydrochalasin B (DHCB). DHCB interferes with actomyosin ring contraction abrogating cytokinesis. This treatment led to rapid cell death in wild-type cells but coincided with increased ploidy in PIDDosome mutant, as well as BCL2 transgenic cells (fig. S5, A to C), in support that the effects caused by PLK4 overexpression are caused by extra centrosomes. PIDD1 was reported to be activated also in the context of IR damage upon ataxia-telangiectasia mutated (ATM)–mediated phosphorylation (*42*), while *Plk4* overexpression may cause DNA damage, e.g., in response to chromosome segregation errors (*43*). To assess if IR and *Plk4* overexpression may act in concert to activate the PIDDosome, we evaluated if (i) loss of *Raidd* affects IR-induced cell death and (ii) ATM inhibition may interfere with PLK4-induced cell killing. Our findings show that loss of Raidd does not affect IR-induced cell death of MPP, but protects from *Plk4* overexpression. In contrast, ATM inhibition reduced IR-induced cell death but had no impact on *Plk4* overexpression (fig. S5D). Dying cells, visualized in IF by using an antibody recognizing cleaved active caspase-3, do not show extra centrioles, indicating their rapid disintegration when the apoptotic program is fully activated (fig. S5, E and F). Together, we conclude that cell death downstream of extra centrosomes in hematopoietic cells is mediated by the PIDDosome and that DNA damage does not contribute to its activation.

### The PIDDosome limits transformation downstream of extra centrosomes

**Fig. 7.**
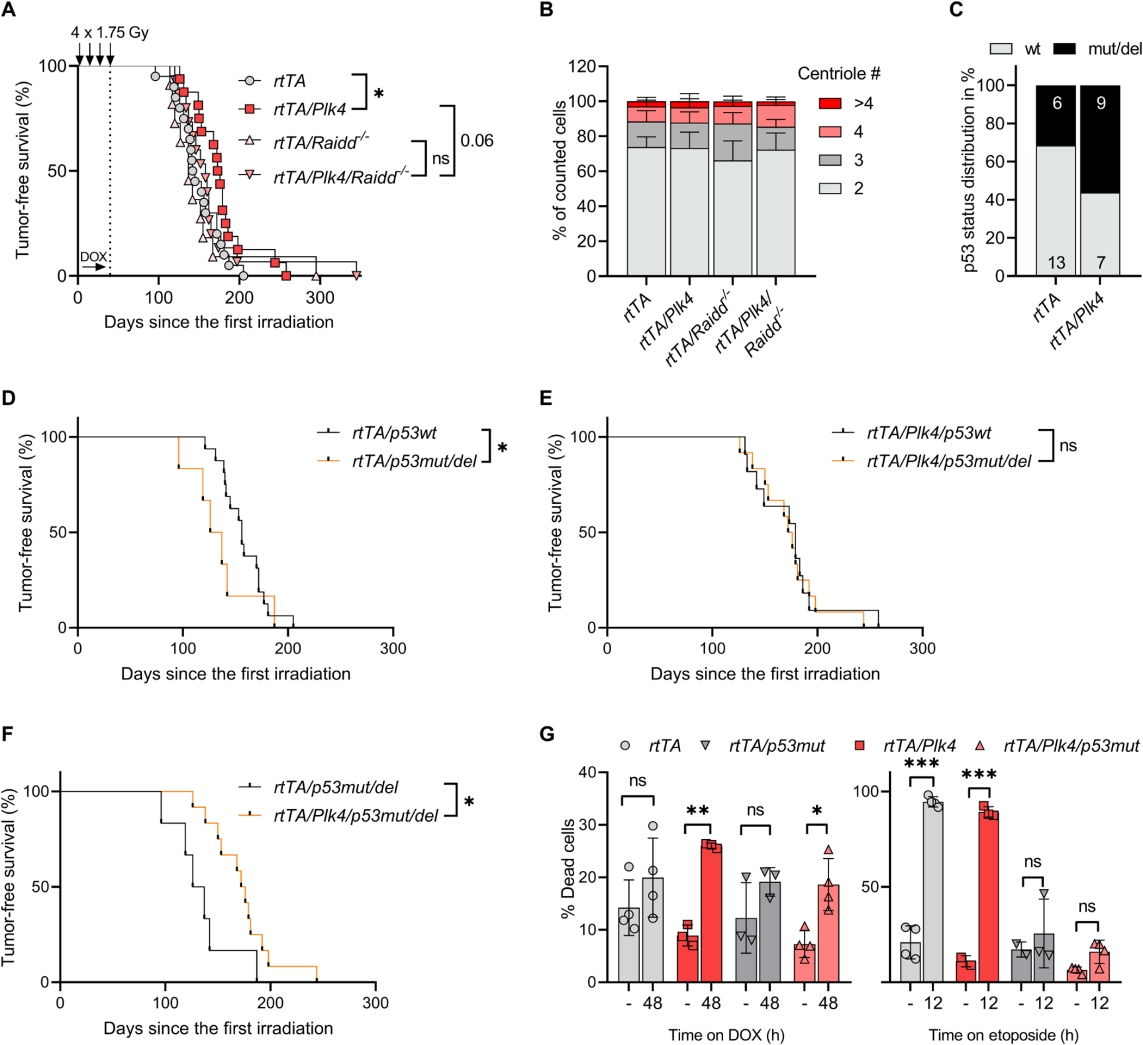
PIDDosome deficiency reverses PLK4-induced delays in IR-driven tumor onset. (**A**) Kaplan-Meier analysis of tumor-free survival. *rtTA* (*n* = 20), median survival 143.5 days; *rtTA/Plk4* (*n* = 16), median survival 174.5 days. Log-rank (Mantel-Cox) *P* = 0.0328 (χ^2^ = 4.554), Breslow-Gehan-Wilcoxon *P* = 0.0232 (χ^2^ = 5.157). *rtTA/Plk4* (*n* = 16) versus *rtTA/Plk4/Raidd^−/−^* (*n* = 15), median survival 174.5 days; *rtTA/Plk4/Raidd^−/−^*, median survival 158 days. Log-rank (Mantel-Cox) *P* = 0.2104 (χ^2^ = 1.569), Breslow-Gehan-Wilcoxon *P* = 0.0644 (χ^2^ = 3.419). (**B**) Tumors were isolated from mice fed with doxycycline-containing food after IR and were analyzed for centrosome amplification by IF using antibodies recognizing CP110 and γ-tubulin (two biological and two technical replicates). (**C**) Quantification of Western blots for p53 and p19ARF to estimate p53 function in respective tumors isolated from terminally ill mice with the indicated genotypes. (**D**) Kaplan-Meier analysis of tumor-free survival of *rtTA* transgenic mice that were defined to have developed p53 wild-type (wt) (*n* = 16) or null (*n* = 6) tumors (median survival 156 versus 131.5 days). Log-rank (Mantel-Cox) *P* = 0.2128 (χ^2^ = 1.552), Breslow-Gehan-Wilcoxon *P* = 0.0332 (χ^2^ = 4.537). (**E**) Tumor-free survival of *rtTA/Plk4* transgenic mice that were defined to have developed p53 wt (*n* = 11) or null (*n* = 12) tumors (median survival 179 versus 174 days). Log-rank (Mantel-Cox) *P* = 0.8194 (χ^2^ = 0.05212), Breslow-Gehan-Wilcoxon *P* = 0.9756 (χ^2^ = 0.0009). (**F**) Tumor-free survival of mice defined as having developed p53 null tumors on an *rtTA* (*n* = 6) or *rtTA/Plk4* background (*n* = 12), median survival 131.5 versus 174 days. Log-rank (Mantel-Cox) *P* = 0.0339 (χ^2^ = 4.5), Breslow-Gehan-Wilcoxon *P* = 0.0118 (χ^2^ = 6.337). *P < 0.05, **P < 0.01, ***P < 0.005. (**G**) Tumor cell lines generated from *rtTA* (four biological replicates), *rtTA/Plk4* (*n* = 1), *rtTA/p53mut/del* (*n* = 1), and *rtTA/Plk4/p53mut/del* (two biological replicates) mice were treated with doxycycline (1 μg/ml) for 48 hours or 1 μM etoposide for 12 hours to induce cell death measured by annexin V/DAPI staining. Up to three technical replicates were performed.

Noting the proapoptotic effects of the PIDDosome in HSPCs, we wondered if this may account for the delay in IR-driven tumorigenesis. Of note, we previously observed that loss of PIDDosome function does not affect IR-driven lymphomagenesis per se (*44*). However, in the context of *Plk4* overexpression, during repeated DNA damage–driven blood cell attrition, loss of RAIDD expression and hence PIDDosome function cancelled out the *Plk4*-induced delay in tumor latency. *Pidd1-*deficient animals also no longer showed a significant delay in tumor formation (Fig. 7A and fig. S6A).

As tumors evolved in the absence of doxycycline, cancer cells isolated from these mice did not show centrosomal abnormalities (Fig. 7B). Nonetheless, we also wondered if the delay in tumor onset may correlate with the level of aneuploidy seen in these cancers. Hence, we subjected tumors of the different genotypes and doxycycline administration protocols to mini-bulk WGS to evaluate their karyotypes (30 cells per tumor). This analysis showed no significant difference between the respective conditions and genotypes (fig. S6, B and C), suggesting that the PIDDosome does not limit aneuploidy in this model system.

To explore the cause for PIDDosome-mediated tumor suppression, we again evaluated p53 pathway status in tumor lysates by Western blot analysis monitoring p53 and p19ARF levels. This analysis revealed that about one-third of all tumors in rtTA mice had lost p53 function (6 of 19), in line with published literature (*37*, *38*). Notably, transient activation of *Plk4* led to a higher number of tumors with p53 pathway inactivation (9 of 16), consistent with the notion that loss of p53 facilitates growth in cells with extra centrosomes (Fig. 7C and fig. S6D). Analyzing tumor latency based on the p53 status, we noted that in *rtTA* mice p53 loss of function accelerates disease, as expected (Fig. 7D). However, this phenotype was lost on the *rtTA/Plk4* transgenic background where both wild-type and p53 mutant tumors showed indistinguishable tumor latency (Fig. 7E). These findings are consistent with the overall delay in disease onset on the *Plk4* transgenic background. Comparing all p53-mutant tumors confirmed that *Plk4* overexpression again triggered a delay in disease onset (Fig. 7F). Together, this suggests that PIDDosome-induced cell death downstream of extra centrosomes may not rely on p53 for tumor suppression and cell death induction might be p53 independent. To test this hypothesis, we exposed tumor-derived cell lines to doxycycline or, for control purposes, etoposide. Cell death was monitored by annexin V/DAPI staining in a flow cytometer. In line with our hypothesis, loss of p53 protected from etoposide killing, but not apoptosis induced by PLK4 overexpression (Fig. 7G).

We conclude that extra centrosomes are not exclusively pro-tumorigenic but can engage mitochondrial apoptosis to delete premalignant clones, delaying transformation induced by DNA damage, even in the absence of p53.

## DISCUSSION

Abnormalities in centrosome number are frequently found in cancer, including leukemia and lymphomas (*16*). Whether these aberrations in centrosome number are cause or consequence of tumorigenesis remains under debate (*45*). Here, we present inaugural evidence that centrosome amplification can even delay cancer formation. This suggests that centrosome abnormalities seen in blood cancer are clearly rather a consequence than a driver of malignant disease. Our results further document that the PIDDosome, a protein complex reported to guard genome integrity by activating p53-induced cell cycle arrest (*25*), can also trigger mitochondrial apoptosis in response to centrosome amplification and that this can delay DNA damage–driven tumorigenesis.

Despite the reported pro-cancerogenic effects of extra centrosomes (*22*), we did not observe any difference in tumor latency in oncogene-driven blood cancer induced by the overexpression of MYC or ABL kinase, when combined with continued *Plk4* transgene expression (Fig. 1, A and E). This, together with our in vitro observations documenting increased cell death in response to *Plk4*-induced centriole amplification (Fig. 2, E, F, and H), led us to postulate that strong oncogene-driven proliferation, known to reduce apoptotic thresholds (*46*), may sensitize cells even further when combined with centrosome amplification. This effect may be caused by increasing rates of CIN or, alternatively, a MYC-induced rewiring of the BCL2 network. Extra centrosomes could then suffice to elicit a death signal that overrides cell cycle arrest, at least in immature B lymphocytes, known to respond preferentially with p53-induced cell death downstream of MYC or ABL oncogene activation (*30*, *47*). In vivo, this may lead to rapid counter-selection of premalignant cells and an increase in the selection pressure for secondary hits. As such, we do not expect that transient activation of *Plk4*, as used in the IR-driven model, has the potential to yield different results. It is worth mentioning that p53 mutation or loss accelerates the onset of *E*μ*MYC* lymphomas (*30*, *35*) and *Abl*-driven transformation (*32*, *33*, *47*) and is found impaired in more than one-third of these tumors, a finding also confirmed here (fig. S1F). However, this seems insufficient to allow survival of tumor cells with extra centrosomes. While shown to facilitate cell growth in the presence of extra centrosomes and aneuploidy tolerance in model epithelial cancer cell lines, p53 deficiency may not suffice to allow survival in the context of MYC or ABL kinase overexpression in hematopoietic cells. Consistently, we failed to observe extra centrosomes in freshly isolated tumor specimens (Fig. 1, D and G) and *Plk4* mRNA expression levels in tumors isolated from *E*μ*MYC* transgenic or *v-Abl*–transduced mice varied substantially straight after isolation (Fig. 1, B and F), despite being readily detectable in vitro (fig. S1D).

Impaired p53 function appears critical to reveal the pro-tumorigenic effects of centrosome amplification downstream of *Plk4* overexpression across all animal models tested so far (*21*, *22*, *24*), but none of those studies introduced oncogenic drivers or IR as alternative tumor-promoting events. As such, the effects of oncogenic stress may simply be dominant over the impact that extra centrosomes may have in the model systems of blood cancer formation tested here. However, it is not yet possible to make a general statement on how oncogene-mediated transformation and centrosome amplification interact in other tissues. This will require the analyses of additional tumor models and oncogenic drivers. For example, previous studies have shown that in the intestine, *APC^min^* mutant mice that experience centrosome amplification show accelerated tumor onset (*22*), while in the skin, chemical carcinogenesis was unaffected by PLK4 overexpression (*20*). These and our studies suggest that the effect of extra centrosomes on cancer can be cell type and context dependent.

In that regard, it is intriguing that transient *Plk4* expression delays onset of T cell lymphomas driven by IR damage (Fig. 4C) in a PIDDosome-dependent manner, as this phenotype is abrogated when *Raidd* (Fig. 7A) or *Pidd1* is lacking (fig. S6A). Moreover, this *Plk4*-induced effect noted in our study was not universal and only became evident when transgene overexpression was induced during the recovery phases from IR-induced myeloablation, but not with chronic transgene activation after the last IR cycle (Fig. 3F). Hematopoietic cell rebound requires high compensatory proliferation of HSPCs and immature progenitor cells, containing pristine malignancy-driving cells (*37*, *38*). These cells may become highly vulnerable to increased centriole counts (Fig. 5), potentially defying CIN-related aneuploidy. Delay in disease onset may be explained by either the effective deletion of HSPCs experiencing high mutational load or prolonged cell cycle arrest allowing for improved DNA damage repair (or a combination of both effects). However, the latter appears less likely, as *p21* facilitates disease onset in response to radiation or ATM deficiency and it is believed that increased cell death rates after DNA damage in the absence of *p21* are tumor protective (*48*). As such, one could argue that increased apoptosis downstream of centrosome amplification exerts a similar protective effect. Consistently, analysis of MPPs from *Caspase-2*-, *Raidd*-, or *Pidd1-*mutant mice, as well as BCL2 transgenic cells, indicates the PIDDosome-dependent induction of mitochondrial apoptosis downstream of *Plk4* overexpression (Fig. 6, B and C). Hence, we hypothesize that the enhanced clearance of HSPCs with extra centrosomes delays disease by reducing the size of the population at risk for transformation during the time when DNA lesions need to be cleared most urgently. Alternatively, sterile inflammation downstream of extra centrosomes (*49**,* *50*), or cell death induction in general during recovery, may increase overall anticancer immunity or render premalignant cells more vulnerable to natural killer (NK) cell attack (*50**,* *51*). However, RAIDD is not involved in sterile inflammation downstream of PIDDosome activation (*52*), yet its loss, similar to loss of PIDD1, neutralized the delay seen in *Plk4* transgenic mice (Fig. 7A and fig. S6A). As such, we favor the role of increased cell death rates of cells at risk to transform due to DNA damage as an explanation for delayed tumor onset. Notably, the 3-MC–induced fibrosarcoma model, also depending on *p53* inactivation for transformation, has been linked to impaired apoptosis induction (*39**,* *53*). Consistent with the IR-driven tumor model, loss of PIDDosome function does not alter 3-MC–driven transformation per se (*54*).

In vitro, BCL2 overexpression proved superior to loss of PIDDosome function in preventing cell death (Fig. 6C). This phenomenon is mainly caused by BCL2 also eliminating spontaneous death in culture. Subtracting this background death actually shows that loss of PIDDosome function is a potent inhibitor of *Plk4*-driven apoptosis (fig. S5D). Hence, we conclude that caspase-2 is a potent initiator of mitochondrial outer membrane permeabilisation (MOMP). How the PIDDosome promotes mitochondrial apoptosis downstream of extra centrosomes remains to be sorted out in detail, but the BH3-only protein BID, a recognized caspase-2 substrate and cell death effector, may be involved (*55*). The notion that *p21* and *Bax* mRNA induction is seen only on a *BCL2* transgenic background in MPPs (Fig. 6, G and H) points potentially toward a simultaneous activation of two effector arms. One of them engages in BCL2-dependent apoptosis directly, and the other relies on MDM2 cleavage and p53 activation for target gene transcription. However, this concept awaits experimental validation.

One may argue that the noted delay in disease onset caused by the PIDDosome is rather modest. However, reducing apoptotic thresholds by removing a single allele of anti-apoptotic *Mcl1* was shown to delay lymphomagenesis in *p53*-mutant mice to a similar degree, while complete removal of *Bclx* had no effect at all (*56*). In contrast, loss of proapoptotic effectors, such as the BH3-only proteins BIM, BAD, or NOXA, has no accelerating impact on IR-driven cancer (*57*). If additional perturbations need to be applied, such as PLK4 overexpression for selective PIDDosome activation, to unravel tumor-suppressive functions for these BH3-only proteins in this disease model, remains to be explored.

In conclusion, existing data explain how centrosome amplification either causes CIN thereby accelerating tumorigenesis (*21*, *22*, *24*) or reduces cellular fitness without having an impact on cancer formation (*20*). The latter fits our observation of grossly normal karyotypes in tumors isolated from *Plk4* transgenic mice exposed to IR or *MYC* hyperactivation, supporting the idea of rapid clearance of (pre)malignant cells by apoptosis, rather than inducing cell cycle arrest or senescence. Our findings identify a new tumor-suppressive effect of centrosome amplification that needs to be overcome during transformation but may be exploited therapeutically. Along this line, it is worth mentioning that human B ALL shows frequent centrosome abnormalities (*58*) and is highly dependent on the centrosome clustering pathway to complete mitosis. This feature may be exploited therapeutically using centrosome clustering inhibitors (*59*). Although we could observe this phenomenon in two different DNA damage–driven cancer models, further studies are needed to determine if similar protective outcomes of centrosome amplification are found in the context of DNA damage–driven malignancies in additional cell types or tissues.

With this, we propose the following model: DNA damage leads to the accumulation of premalignant clones that sample different mutations, most notably in p53, eventually allowing full transformation. If centrosomes are amplified during this phase, this alters apoptotic priming and a wave of PIDDosome-dependent apoptosis occurs, which eradicates potential premalignant clones, including those lacking p53. This causes a transient tumor-suppressive effect resulting in delayed disease onset. This phenomenon may also be exploited during cancer therapy, combining DNA-damaging agents with compounds promoting defects in cytokinesis, leading to centrosome amplification, such as Aurora or MPS1 kinase inhibitors, known to activate the PIDDosome (*25*). Along this line, a recent preprint documents heightened cell death susceptibility of PLK4-overexpressing ovarian cancer cells toward carboplatin treatment and improved overall survival and therapy responsiveness of patients showing centrosome amplification (*60*). Together, these findings ask for a reassessment of the role of extra centrosomes in tumorigenesis and as markers of disease progression.

## MATERIALS AND METHODS

### Mice

Animal experiments were conducted in consideration of the Austrian Legislation BMWF: 66.011/0159-V/3b/2019 and 2021-0.607.468. All mouse strains were maintained on a C57BL/6N genetic background. The *rtTA* transgene and the *TET-Plk4* allele (*22*) were both maintained in a hemizygous state on a wild-type, *E*μ*MYC*-transgenic (*28*), *Pidd1-* or *Raidd*-deficient background. This allowed the generation of semi-blinded cohorts and the use of littermate controls. In some instances, EGFP-*Centrin1* was crossed in as an additional reporter gene (*61*). Ventilated cages with nesting material and a 12:12-hour light:dark cycle served as housing conditions. Doxycycline-containing food (Sniff GmbH, 625 mg/kg) was fed for the indicated periods of time. Mice were analyzed with an age of 8 to 16 weeks. Thymic lymphomas were induced by a weekly dose of 1.75 Gy over 4 weeks. Pro/pre-B ALL was induced by neonatal injection (100 μl) of *v-Abl* retrovirus subcutaneously in the neck fold within 48 hours after birth (*29*). Muscular sarcoma formation was induced by a single 100-μl intramuscular injection of 1 mg of 3-MC per mouse (Sigma-Aldrich, Vienna, Austria), dissolved in sesame oil. Mice treated with sesame oil alone (vehicle) served as a control.

### Cell culture

MethoCult colony formation assays (STEMCELL Technologies, SF H4436) were performed by isolating fetal liver (E14.5) and seeding 10^3^ cells per milliliter into 35-mm cell culture dishes. Cells were cultured with and without doxycycline (1 μg/ml) (Sigma-Aldrich, D-9891) in M-CSF (PeproTech, 315-02)– or G-CSF (PeproTech, 250-05)–containing medium (Iscove′s modified Dulbecco′s medium, Sigma-Aldrich). After 10 days, colonies were counted in a blinded fashion.

Thymocytes were isolated from thymic tissue, mashing it through 70-μm strainers (Falcon 352350) and culturing the single-cell suspension in RPMI 1640 medium (Sigma-Aldrich, R0883), supplemented with 10% fetal calf serum (FCS) (Sigma-Aldrich, F0804), 2 mM l-glutamine (Sigma-Aldrich, G7513), penicillin (100 U/ml) and streptomycin (100 μg/ml) (Sigma-Aldrich, P0781), and 50 μM 2-mercaptoethanol (2-ME) (Sigma-Aldrich, M3148). Tumor cell lines were generated accordingly, using thymic tumor tissue. Outgrowth of tumor cell lines started after about 1 week.

The same medium, additionally containing 1 μM β-estradiol and 5% supernatant of FLT3L-expressing B16-melanoma cells, was used to generate HSPC-like MPP cultures. Opti-MEM (Gibco, catalog no. 31985070) supplemented with 10% FCS, 2 mM l-glutamine, penicillin (100 U/ml), streptomycin (100 μg/ml), 50 μM 2-ME, and 2% supernatant of SCF-producing WEHI-231 cells was used for SMPs. In general, cells were cultured at 37°C in a humidified atmosphere containing 5% CO_2_.

### Pro-B cell culture

To culture pro-B cells outside of the body, a specific group of cells (B220^lo^CD19^+^IgD^−^IgM^−^) was FACS (fluorescence-activated cell sorting)–sorted from the bone marrow of 5-week-old mice, totaling 1 × 10^6^ cells. Approximately one-third of these cells were subjected to cell cycle analysis and centriole count, while the remaining cells were placed in 96-well plates in triplicate in B cell medium. This medium consists of Dulbecco’s modified Eagle’s medium (DMEM) (Thermo Fisher Scientific) medium supplemented with 10% FCS, 2 mM l-glutamine, 0.055 mM 2-ME, 10 mM Hepes, 1 mM sodium pyruvate, penicillin (100 U/ml), and streptomycin (100 μg/ml) (GIBCO). Additionally, interleukin-7 (IL-7) (10 ng/ml) (PeproTech) was added to the medium. A separate 96-well was prepared for each day of analysis (3, 5, and 7). The cells were harvested by pipetting, washed once with phosphate-buffered saline (PBS), and then evenly distributed for cell cycle analysis and IF for centriole enumeration.

### Generation of HoxB8 cell lines

Bone marrow was isolated from 8- to 16-week-old mice and cultured for 3 days in Opti-MEM (Gibco, catalog no. 31985070) supplemented with 10% FCS, 2 mM l-glutamine, penicillin (100 U/ml), streptomycin (100 μg/ml), 50 μM 2-ME, IL-3 (10 ng/ml) (lot #120948 C2013, PeproTech), IL-6 (20 ng/ml) (lot #090850 A3013, PeproTech), and 2% supernatant of SCF-producing WEHI-231 cells. Subsequently, 2 × 10^5^ cells were used for spin infection with Hoxb8-encoding virus and Metafectene (1:1000, Biontex, T020) by three cycles of 30-min spin-down at 37°C (400*g*, vortex in between). Cells were then split into either HSPC-like FLT3-dependent MPP- or SMP-specific medium to generate the respective cell line. To generate the Bcl2 transgenic MPPs, 2 × 10^5^ cells were used for spin infection with retrovirus produced by transiently cotransfecting 293T cells with Addgene-derived constructs for VSVG, Hit60, and either pMIG or pMIG-Bcl2.

### Flow cytometry and cell sorting

Single-cell suspensions were generated by flushing both femora to isolate sufficient bone marrow with a 27-gauge × ^1^/_2_ (HENKE-JECT) needle or mashing spleen, thymus, or lymph nodes through a 70-μm filter (Corning, 352350). Peripheral blood was isolated from the vena mandibularis, and erythrocyte lysis was performed by incubation with 155 mM NH_4_Cl, 10 mM KHCO_3_, and 0.1 mM EDTA (pH 7.5) for 3 min at room temperature. Samples were stained with the antibodies indicated below in PBS with 2% fetal bovine serum (FBS) (Gibco, 10270-106) for 10 min at 4°C. Flow cytometric analysis was performed using the LSR Fortessa or cell-sorted via FACSAria III (both BD). The data were analyzed using FlowJo v10 software. The following antibodies were used: Ter119 Bio (eBioscience 13-5921-75), CD3 Bio (eBioscience 13-0031-75, 145-2C11), CD11b Bio (eBioscience 13-0112-75, M1/70), Gr1 Bio (eBioscience 13-5931-75, RB6-8C5), NK1.1 Bio (BioLegend 108704, PK136), streptavidin BV605 (BioLegend 405229), c-kit phycoerythrin (PE)/Cy7 (BioLegend 105814, 2B8), Sca1 allophycocyanin (APC) (BioLegend 108107, D7), CD4 APC (BioLegend 100530, RM4-5), CD8 PE (BioLegend 100708, 53-6.7), IgD peridinin chlorophyll protein (PerCP)/Cy5.5 (BioLegend 405710, 11-26C.2A), CD19 PE (BioLegend 115506, 115540, 6D5), IgM APC (BioLegend 406509, RMM-1), B220 PE/APC-Cy7 (BioLegend 103208, 103224 RA3-6B2), CD43 PE (BD 553271, S7), and CD25 PE (BioLegend 101904, 3C7).

### Viability assays and caspase-3 activity assessment

Single-cell suspensions were harvested and stained with annexin V–Alexa 647 (lot: B311285, BioLegend, 1:1000), in annexin V binding buffer (PNN1001, Invitrogen) and DAPI (4′,6-diamidino-2-phenylindole; 1 μg/ml) (Sigma-Aldrich, D-9542). Subsequently, the percentage of apoptotic/dead cells was analyzed by flow cytometry. To analyze caspase-3 activity, 4 μM caspase-3 substrate (Biotium, 10406) was added to DMEM complete medium as a supplement. Fluorescence was measured over time using the IncuCyte S3 system. Analysis was performed using ×10 magnification (phase confluency/red object area in micrometers). To access sub-G_1_ or >4*n* population, cells were fixed in 1-ml ethanol (70%) and stored at −20°C for a minimum of 60 min. Subsequently PI staining (4 μg/ml; Sigma-Aldrich) was performed for 30 min at 37°C in the presence of ribonuclease (RNase) A. Drug treatments also included ATM inhibitor KU-55933 (MedChemExpress) and DHCB (Sigma-Aldrich, D1641) in concentrations indicated in the respective experiments.

### Gene expression analyses by qRT-PCR

RNA was isolated from respective snap-frozen cell pellets according to the manufacturer’s instructions of the Qiagen RNeasy Mini Kit (74104) and RNase-Free DNase Set (79254). iScript cDNA Synthesis kit (1708890) was used to generate cDNA, using 100 ng of each sample. Quantitative reverse transcription polymerase chain reaction (qRT-PCR) was performed with the StepOnePlus System (Applied Biosystems). 2× SYBR Green qPCR Master Mix (BioTool, B21203) was used to measure the expression levels of *Plk4* mRNA, normalized to the *Hprt*-housekeeping gene. Primers: (5′–3′): *Plk4*_Fw, GGAGAGGATCGAGGACTTTAAGG; *Plk4*_Rv, CCAGTGTGTATGGACTCAGCTC; Hprt_Fw, GTCATGCCGACCCGCAGTC; *Hprt_Rv*, GTCCTTCCATAATAGTCCATGAGGAATAAAC; BAX_Fw, TGAAGACAGGGGCCTTTTTG; BAX_Rv, AATTCGCCGGAGACACTCG; p21_Fw, AATTGGAGTCAGGCGCAGAT; p21_Rv, CATGAGCGCATCGCAATCAC.

### Immunoblotting

Lysis of the cell pellets was performed in 50 mM tris (pH 8.0), 150 mM NaCl, 0.5% NP-40, 50 mM NaF, 1 mM Na_3_VO_4_, 1 mM phenylmethylsulfonyl fluoride (PMSF), one tablet protease inhibitors (EDTA free, Roche) per 10 ml, and deoxyribonuclease (DNase; 30 μg/ml) (Sigma-Aldrich). After Western blotting and wet transfer in ethanol-based transfer buffer onto nitrocellulose membranes (Cytiva), proteins were detected by chemoluminescence (Advansta, K-12049-D50) using rat anti–caspase-2 (11B4, Enzo Life Science), rabbit anti-p19ARF (sc-32748, Santa Cruz Biotechnology), mouse anti-p53 (1C12, Cell Signaling), rabbit anti-p21 (ab7960), mouse anti-HSP90 (sc-13119, Santa Cruz Biotechnology), rabbit anti–glyceraldehyde-3-phosphate dehydrogenase (GAPDH) (14C10, Cell Signaling), and mouse anti-hBCL2 (clone S100). Goat anti-rabbit Ig/horseradish peroxidase (HRP) (Dako, P0448) or rabbit anti-mouse Ig/HRP (Dako, P0161) was used as secondary reagent, respectively.

### Immunofluorescence

Coverslips were poly-l-lysine–coated (Sigma-Aldrich, 1:10 in aqua dest) for 30 min at 37°C. The cells where then plated in low volume (ranging from 1 to 5 μl; approximately 25,000 cells/μl) on a coated coverslip and incubated for 15 min at 37°C. Subsequently, the cells were fixed with 4% paraformaldehyde for 10 min at room temperature and washed 3 × 10 min with PBS containing 0.1% Triton X-100. After permeabilization (0.5% Triton, 10 min at room temperature), cells were blocked in 5% bovine serum albumin (GE Healthcare), 0.2% Tween 20 (National Diagnostics), and PBS for 1 hour at room temperature. The same conditions were used for incubation with the primary antibodies, rabbit anti-CP110 (Proteintech, 12780-1-AP, 1:600), mouse anti–γ-tubulin (Sigma-Aldrich, 1:300), and rabbit anti–cleaved caspase 3 (Cell Signaling, 5A1E). The cells were rewashed 3 × 10 min and stained with the secondary antibodies for 1 hour at room temperature in blocking buffer, anti-rabbit Alexa Fluor 568 (Invitrogen, A11031, 1:1000), and anti-mouse Alexa Fluor 647 (Invitrogen, A21236, 1:1000). Cells were rewashed 3 × 10 min, with the first washing step containing DAPI (1 μg/ml) for nuclear staining. Subsequently, mounting was performed in Mowiol mounting solution. Pictures were acquired using an SP5 confocal microscope (Leica, 100×). Centrosome quantification was performed using ImageJ Fiji.

### Whole genome mini-bulk sequencing

Tumor cells were isolated from thymi of tumor-baring mice. Cells were suspended in buffer containing tris-HCl, sucrose, calcium chloride, magnesium acetate, EDTA, dithiothreitol (DTT), and Triton X-100. Tissue and nuclei separation was then performed by passing solutions through a 70-μm filter using syringe plunger, and nuclei were collected by centrifugation. The resulting nuclei pellet was suspended in a solution of PBS with bovine serum albumin, Hoechst 33358, and PI. For library preparation, approximately 30 nuclei per tumor were sorted into a single well, following a method described in a previous study (*62*). The sequencing step was performed using a NextSeq 500 instrument (Illumina, San Diego, CA). To align the sequencing data, the murine reference genome (GRCm38) was used in combination with the Bowtie2 (v2.2.4) software (*63*). Copy number variation analysis was conducted using the AneuFinder R package (v1.10.1) (*64*). The libraries underwent several processing steps, including GC correction, removal of artifact-prone regions, and mappability checks. The edivisive copy number calling algorithm was applied with a bin size of 1 Mb, and the modal copy number state was determined based on the expected ploidy state. The breakpoint detection parameter was set to 0.9. The aneuploidy score for each library was calculated as the average absolute deviation from the expected euploid copy number per bin.
