# Supplementary material for: Extra centrosomes delay DNA damage–driven tumorigenesis
Source: Sci Adv. 2024 Mar 29;10(13):eadk0564. doi: 10.1126/sciadv.adk0564 (PMC10980279; doi:10.1126/sciadv.adk0564)
Supplement: Supplementary file 1 — Figs. S1 to S6 [file sciadv.adk0564_sm.pdf]

Science Advances
AAAS

Supplementary Materials for

Extra centrosomes delay DNA damage–driven tumorigenesis

Vincent Z. Braun *et al.*

Corresponding author: Andreas Villunger, andreas.villunger@i-med.ac.at

Sci. Adv. **10**, eadk0564 (2024)
DOI: 10.1126/sciadv.adk0564

This PDF file includes:

Figs. S1 to S6

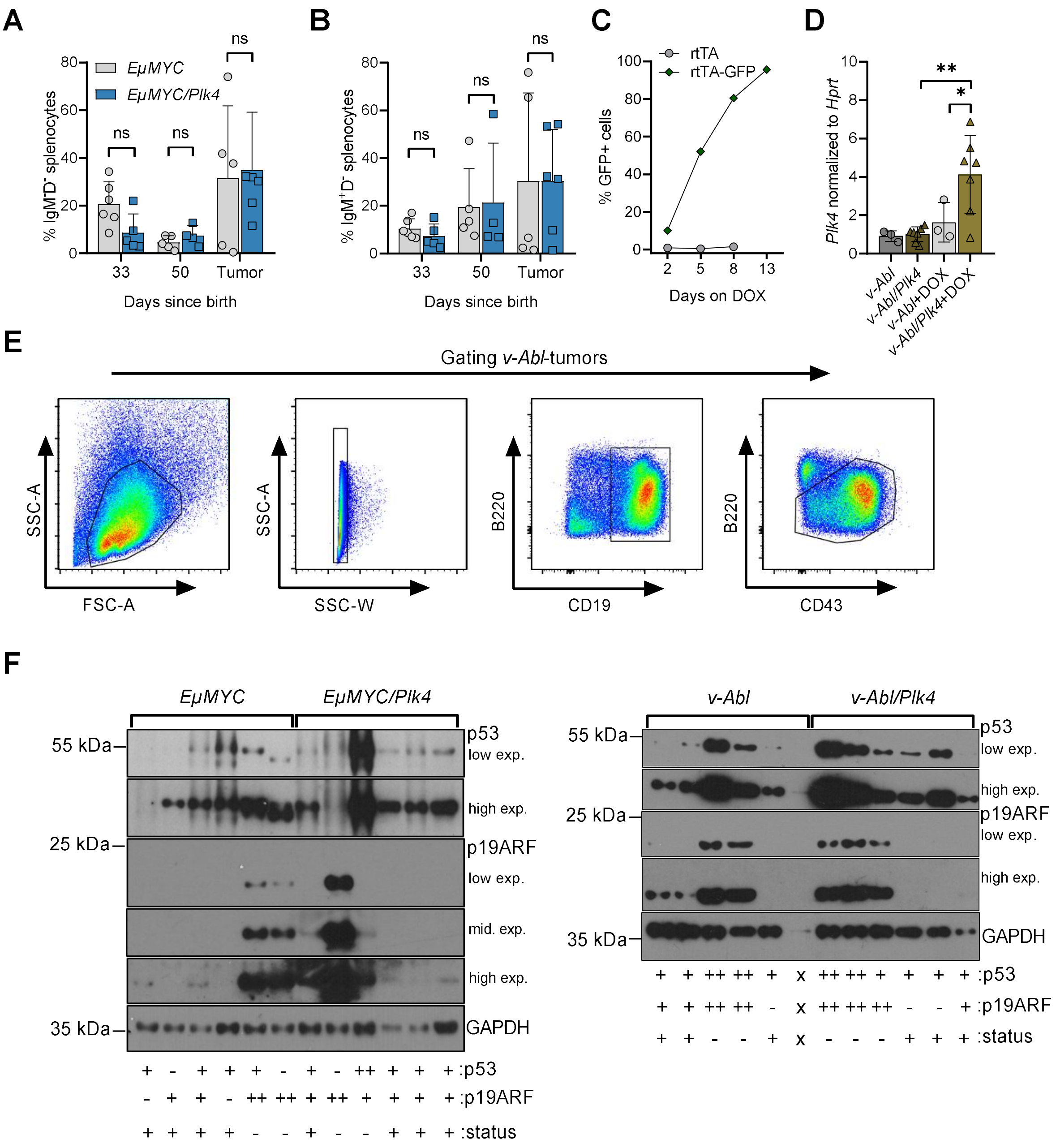
A
EμMYC
EμMYC/Plk4
% IgM-D- splenocytes
ns
Days since birth
33
50
Tumor
B
% IgM+D- splenocytes
C
rtTA
rtTA-GFP
% GFP+ cells
Days on DOX
D
Plk4 normalized to Hprt
v-Abl
v-Abl/Plk4
v-Abl+DOX
v-Abl/Plk4+DOX
E
Gating v-Abl-tumors
SSC-A
FSC-A
SSC-W
B220
CD19
CD43
F
EμMYC
EμMYC/Plk4
55 kDa
25 kDa
35 kDa
p53
low exp.
high exp.
p19ARF
mid. exp.
GAPDH
+ - + + + - + - ++ + + + :p53
- + + - ++ ++ + ++ + + + + :p19ARF
+ + + + - - + - - + + + :status
v-Abl
v-Abl/Plk4
+ + ++ ++ + x ++ ++ + + + + :p53
+ + ++ ++ - x ++ ++ ++ - - + :p19ARF
+ + - - + x - - - + + + :status

Supplementary Figure 1: *Plk4* overexpression does not impact *EμMYC* or *v-Abl* driven lymphoma. (A) Percentage of pro/pre- and **(B)** immature IgM$^+$ B cells found in the peripheral blood of premalignant animals on day 33 (5 days on doxycycline; *EμMYC* n=6, *EμMYC/Plk4* n=4) or day 50 (*EμMYC* n=5, *EμMYC/Plk4* n=4), and in MYC-driven tumors (*EμMYC* n=6, *EμMYC/Plk4* n=5). **(C)** Percentage of CD19$^+$ B cells in the peripheral blood of neonatal mice expressing a doxycycline-responsive GFP reporter. **(D)** *Plk4* mRNA expression of tumor cell lines established from *R26rtTA* and *R26rtTA/TET-Plk4* transgenic mice infected with *v-Abl*-encoding retrovirus. *v-Abl* (n=3), *v-Abl/Plk4* (n=7). **(E)** Gating strategy for the characterization of acute lymphatic leukemias derived from *R26rtTA* and *R26rtTA/Plk4* transgenic mice infected neonatally with *v-Abl*-encoding retrovirus. **(F)** Western blot analysis of p53 and p19ARF protein levels in *MYC*-driven lymphomas and *v-Abl*-driven leukemias to assess p53 functional state in tumors isolated from terminally ill *EμMYC* (n=6), *EμMYC/Plk4* (n=6), *v-Abl* (n=5) and *v-Abl/Plk4* (n=6) mice. Data is shown as mean ± SD. Pro B cell culture data were statistically tested by Sidak's multiple comparisons test. mRNA expression in *v-Abl* tumor cells were tested by Tukey's multiple comparisons test. *P < 0.05, **P < 0.01, ***P < 0.005, ns= not significant.

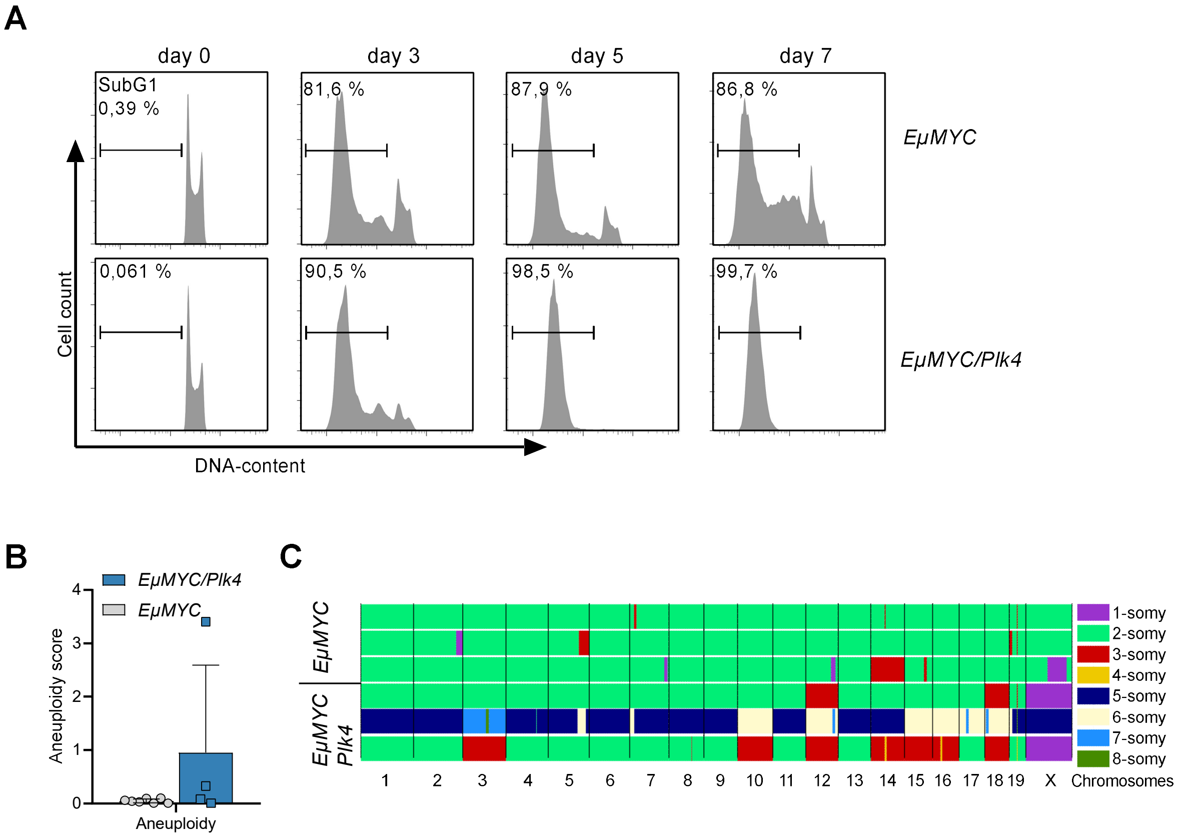

Supplementary Figure 2: *Plk4* overexpression promotes cell death but has minor impact on aneuploidy levels. (A) Representative histograms defining the subG1 population in pro B cells isolated from *EμMYC* and *EμMYC/Plk4* mice cultured for up to 7 days. **(B)** Aneuploidy score and **(C)** representative karyograms created by mini-bulk sequencing of tumors (30 cells/tumor) derived from *EμMYC* (n=7) and *EμMYC/Plk4* (n=4) transgenic mice Data is shown as mean ± SD. Aneuploidy data were statistically tested by unpaired t test with Welch's correction.

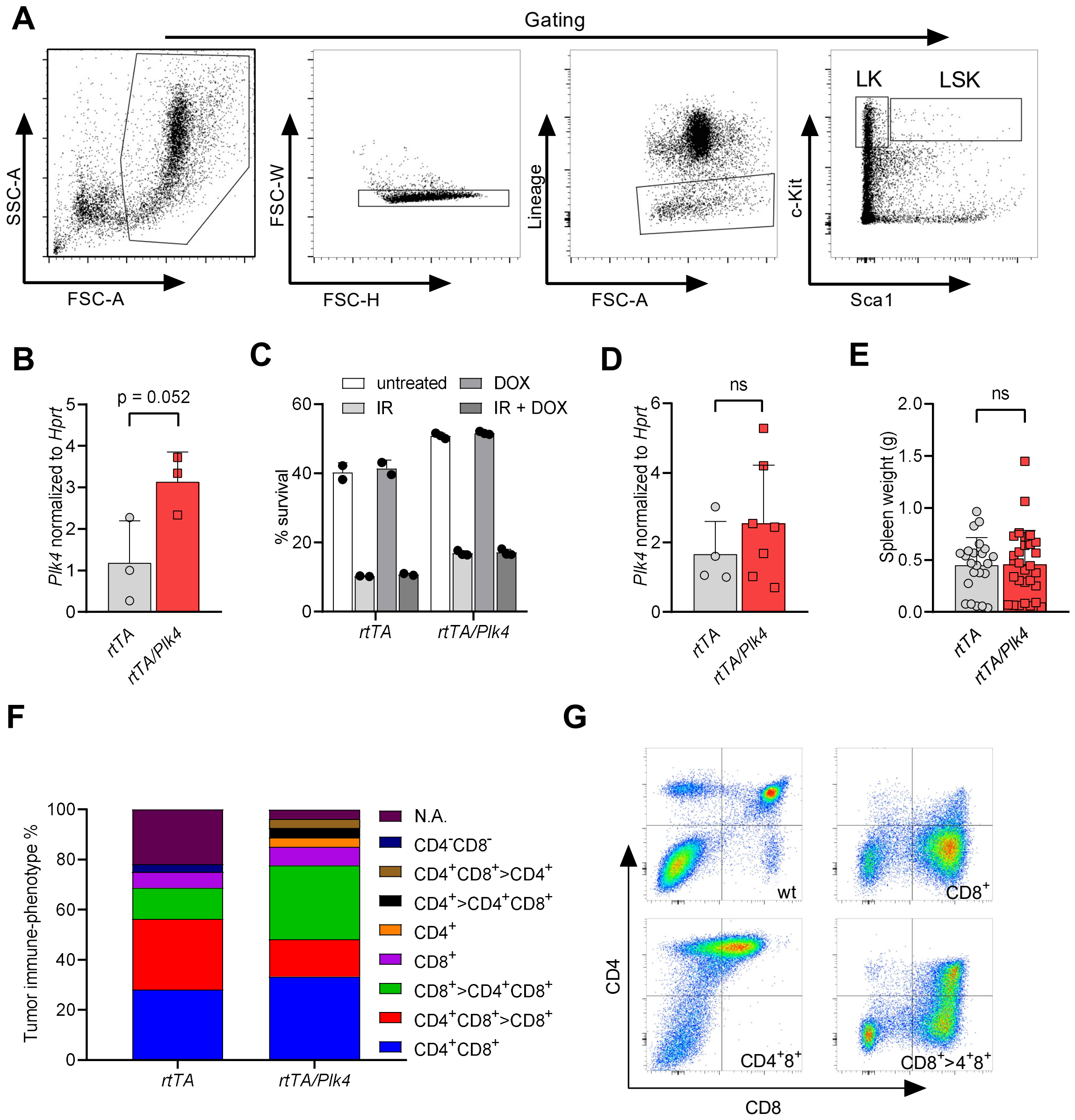
A
Gating
SSC-A
FSC-A
FSC-W
FSC-H
Lineage
FSC-A
LK
LSK
c-Kit
Sca1
B
p = 0.052
Plk4 normalized to Hprt
rtTA
rtTA/Plk4
C
untreated
IR
DOX
IR + DOX
% survival
rtTA
rtTA/Plk4
D
ns
Plk4 normalized to Hprt
rtTA
rtTA/Plk4
E
ns
Spleen weight (g)
rtTA
rtTA/Plk4
F
Tumor immune-phenotype %
N.A.
CD4⁻CD8⁻
CD4⁺CD8⁺>CD4⁺
CD4⁺>CD4⁺CD8⁺
CD4⁺
CD8⁺
CD8⁺>CD4⁺CD8⁺
CD4⁺CD8⁺>CD8⁺
CD4⁺CD8⁺
rtTA
rtTA/Plk4
G
wt
CD8⁺
CD4⁺8⁺
CD8⁺>4⁺8⁺
CD4
CD8

Supplementary Figure 3: *Plk4* overexpression does not affect hematopoietic recovery after IR, nor tumor immune phenotypes. (A) Representative dot plots documenting the gating strategy used to isolate or quantify LSK and LK cells by flow cytometry. **(B)** *Plk4* mRNA expression analysis of *ex vivo* cultured thymocytes treated with 1 µg/ ml doxycycline for 24h, normalized to *Hprt*. *R26rtTA* (n=3), *R26rtTA/Plk4* (n=3). **(C)** Thymocytes were isolated from *R26rtTA* (n=2) and *R26rtTA/Plk4* (n=3) mice. Cells were exposed to a single IR-dose of 10 Gy of IR and cultured ± 1 µg/ml doxycycline for 24h. Viability was assessed by Annexin V and Topro-3 staining by flow cytometric analyses. **(D)** *Plk4* mRNA expression in freshly isolated tumors from *R26rtTA* (n=4) and *R26rtTA/Plk4* (n=7) mice. *Hprt* expression was assessed in parallel for normalization. **(E)** Assessment of the relative tumor burden by spleen weight in *R26rtTA* (n=23) and *R26rtTA/Plk4* (n=29) mice. **(F)** Thymic lymphomas were subjected to immune phenotyping using fluorochrome-labelled anti-CD4- and anti-CD8-specific antibodies. **(G)** Representative dot-plots of single cell suspensions stained with anti-CD4 or anti-CD8-specific antibodies to define single positive (SP), double positive (DP) or oligoclonal/mixed tumors (e.g. $CD4^{+}CD8^{+} > CD8^{+}$). Significance was tested by unpaired t-test for *Plk4* mRNA expression and spleen weight data. Cell death data is shown as mean ± SD and was tested for significant differences using the Sidak's multiple comparisons test. *P < 0.05, **P < 0.01, ***P < 0.005, ns= not significant.

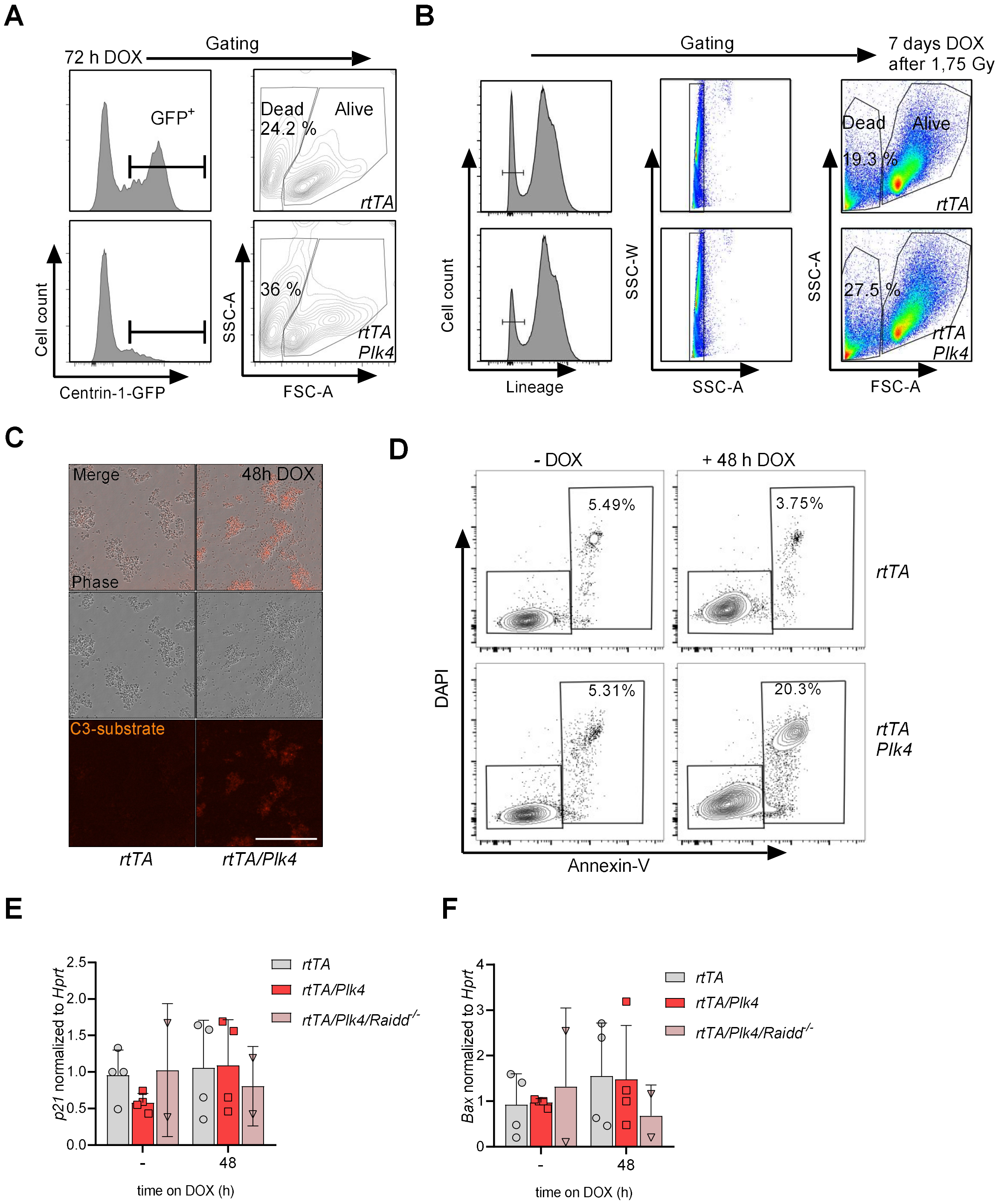

A
72 h DOX
Gating
GFP+
Dead
24.2 %
Alive
rtTA
36 %
rtTA
Plk4
Cell count
Centrin-1-GFP
SSC-A
FSC-A
B
Gating
7 days DOX
after 1,75 Gy
Cell count
Lineage
SSC-W
SSC-A
Dead
Alive
19.3 %
rtTA
27.5 %
rtTA
Plk4
SSC-A
FSC-A
C
Merge
48h DOX
Phase
C3-substrate
rtTA
rtTA/Plk4
D
- DOX
+ 48 h DOX
5.49%
3.75%
rtTA
5.31%
20.3%
rtTA
Plk4
DAPI
Annexin-V
E
p21 normalized to Hprt
2.5
2.0
1.5
1.0
0.5
0.0
-
48
time on DOX (h)
rtTA
rtTA/Plk4
rtTA/Plk4/Raidd-/-
F
Bax normalized to Hprt
4
3
2
1
0
-
48
time on DOX (h)
rtTA
rtTA/Plk4
rtTA/Plk4/Raidd-/-

Supplementary Figure 4: ***Plk4*** **overexpression limits the viability of hematopoietic stem and progenitor cells. (A)** Representative histograms identifying Centrin-1-GFP^{+} MPP cells of the indicated genotypes. GFP-positive cells were co-cultured with GFP-negative wild type cells for 24h, 48 and 72h in the presence of doxycycline (1 µg/ml). Forward/side-scatter analysis was used to define the percentage of viable/dead cells in experiments shown in Fig. 5D and quantified in Fig. 5F. **(B)** Representative histograms of Lin-negative bone marrow cells isolated 7 days after exposure to a single dose of IR (1.75 Gy) from animals of the indicated genotypes that were kept on doxycycline-containing food up to 7 days. The Lin-negative fraction was subjected to doublet-exclusion and subsequently analyzed by forward/side-scatter separation to estimate viability. **(C)** Representative image of time dependent Caspase-3 substrate processing in multipotent hematopoietic progenitors (MPP) after doxycycline treatment measured over time by the IncuCucyte system, quantified in Fig. 6A. scale bar 300 µm. **(D)** Representative dot-plots of Annexin V/PI staining of MPP exposed to doxycycline for 24h or 48h, quantified in Fig 6B. **(E)** *p21* and **(F)** *Bax* mRNA expression was quantified in MPPs established from *R26rtTA* (n=4), *R26rtTA/Plk4* (n=4) and *R26rtTA/Plk4/Raidd*$^{-/-}$ (n=2) mice. RNA was isolated before or after 48h of doxycycline treatment (1µg/ml; n=2). Data is shown as mean ± SD and tested by Sidak's multiple comparisons test. *P < 0.05, **P < 0.01, ***P < 0.005, ns= not significant.

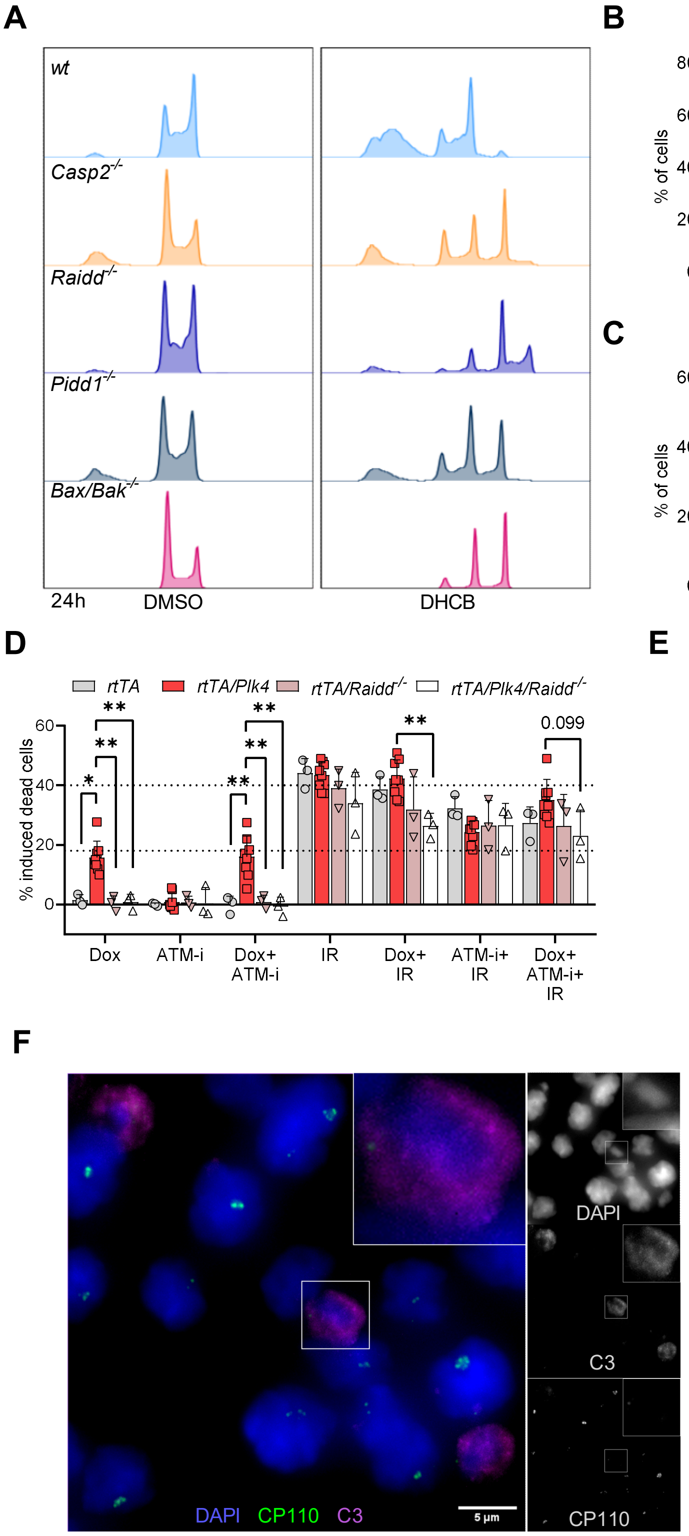
A
wt
Casp2-/-
Raidd-/-
Pidd1-/-
Bax/Bak-/-
24h
DMSO
DHCB
B
% of cells
subG1 DMSO
subG1 DHCB
C
>4n DMSO
>4n DHCB
0.09
D
rtTA
rtTA/Plk4
rtTA/Raidd-/-
rtTA/Plk4/Raidd-/-
% induced dead cells
Dox
ATM-i
Dox+ ATM-i
IR
Dox+ IR
ATM-i+ IR
Dox+ ATM-i+ IR
0.099
E
% cl. casp3+ cells
rtTA/Plk4
rtTA/Plk4+DOX
rtTA/Plk4/Raidd-/-
rtTA/Plk4/Raidd-/- +DOX
rtTA/Plk4/Pidd1-/-
rtTA/Plk4/Pidd1-/- +DOX
F
DAPI CP110 C3
5 µm
DAPI
C3
CP110

Supplementary Figure 5: PIDDosome loss protects from polyploidy-induced cell death but not IR damage. (A) Representative histograms of HoxB8-immortalized bone-marrow derived progenitor cells of different genotypes, treated with 5 µM DHCB to induce cytokinesis failure and centrosome accumulation. DNA content and cell death were assessed in parallel by propidium iodide staining of ethanol-fixed cells. **(B)** Percentage of subG1 cells 24h after treatment with DHCB or DMSO (wt n=12, *Casp2*$^{-/-}$ n=8, *Raidd*$^{-/-}$ n=7, *Pidd1*$^{-/-}$ n=7, *Bax/Bak*$^{-/-}$ n=2). **(C)** Percentage of cells shown in (A) with a DNA content > 4n. **(D)** HoxB8-immortalized progenitor cells of the indicated genotypes were exposed to doxycycline in the absence or presence of 500 nM ATM-KU55933 (ATMi). After 40h, cells were exposed to a single dose of IR (0.2 Gy). Cell death was quantified after an additional 5h by propidium iodide and Annexin V staining by flow cytometric analysis. Treatment-induced cell death over background is displayed. *R26rtTA* (n=3), *R26rtTA/Plk4* (n=9), *R26rtTA/Raidd*$^{-/-}$ (n=3), *R26rtTA/Plk4/Raidd*$^{-/-}$ (n=3). **(E)** Quantification of caspase 3 positive cells per field relative to the overall cell count per field of *R26rtTA/Plk4* (n=2), *R26rtTA/Plk4/Raidd*$^{-/-}$ (n=1) and *R26rtTA/Plk4/Pidd1*$^{-/-}$ (n=2). 2-3 technical replicates were performed. **(F)** Representative IF of (E) showing cl. caspase 3, DAPI and CP110.

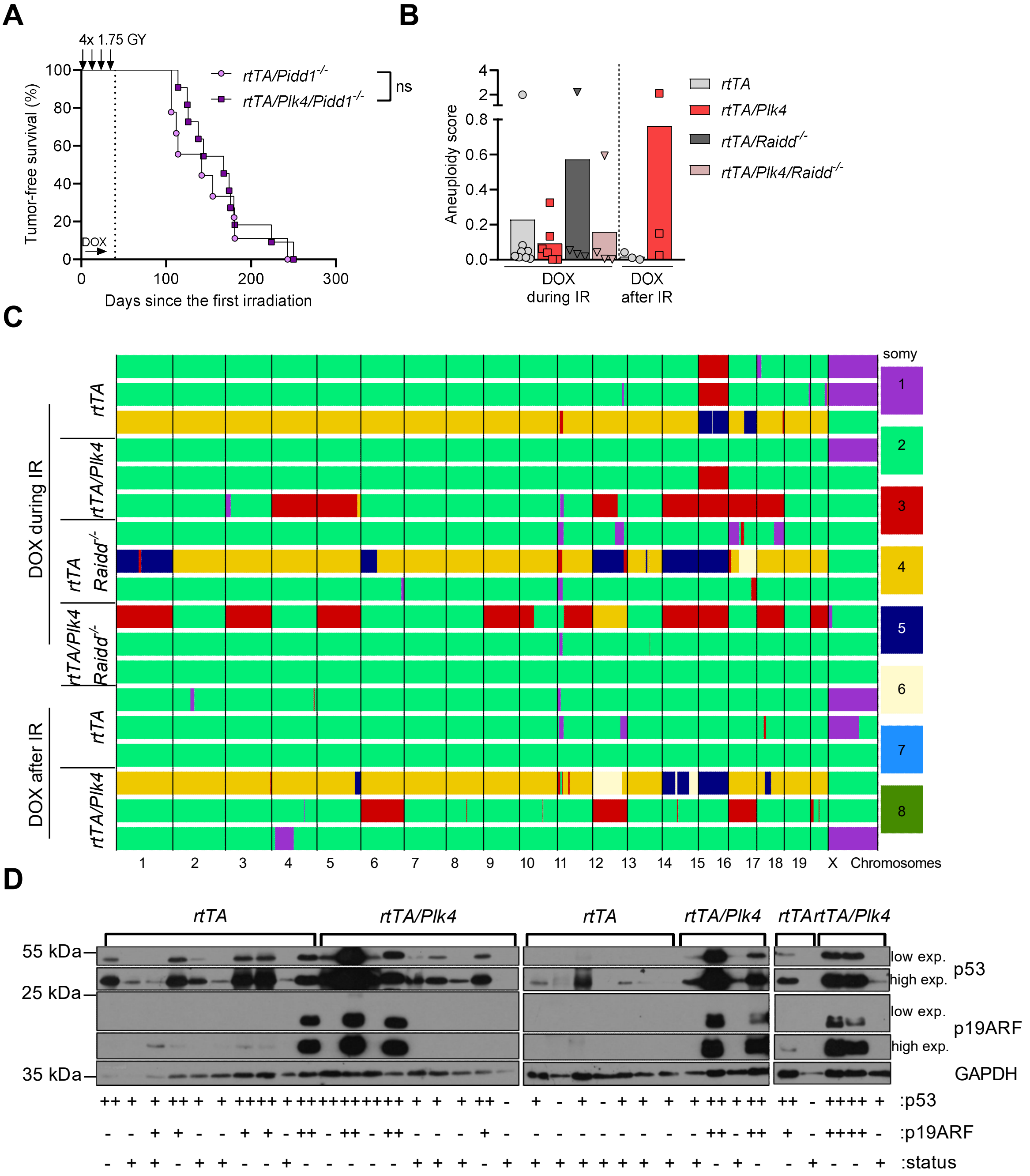

A
4x 1.75 GY
Tumor-free survival (%)
100
80
60
40
20
0
DOX
0
100
200
300
Days since the first irradiation
rtTA/Pidd1-/-
rtTA/Plk4/Pidd1-/-
ns
B
Aneuploidy score
4
2
0.8
0.6
0.4
0.2
0.0
DOX during IR
DOX after IR
rtTA
rtTA/Plk4
rtTA/Raidd-/-
rtTA/Plk4/Raidd-/-
C
DOX during IR
DOX after IR
rtTA
rtTA/Plk4
rtTA Raidd-/-
rtTA/Plk4 Raidd-/-
somy
1
2
3
4
5
6
7
8
1 2 3 4 5 6 7 8 9 10 11 12 13 14 15 16 17 18 19 X Chromosomes
D
rtTA
rtTA/Plk4
55 kDa
25 kDa
35 kDa
low exp.
high exp.
p53
p19ARF
GAPDH
:p53
:p19ARF
:status

Supplementary Figure 6: ***Plk4*****-overexpression does not affect aneuploidy in IR-driven cancers. (A)** Kaplan-Meier analysis of tumor free survival of *rtTA* and *rtTA/Plk4* transgenic mice lacking or expressing *Pidd1*. *rtTA Pidd1*$^{-/-}$ (n = 10), *rtTA/Plk4/Pidd1*$^{-/-}$ (n = 12). Logrank (Mantel-Cox) p = 0,476 (χ 2 = 0,5066), Breslow–Gehan–Wilcoxon p = 0,2992 (χ 2 = 1,078). **(B)** Aneuploidy score of tumor cells isolated from mice fed with doxycycline-containing food during or after IR by whole genome sequencing of mini-bulks (30 cells/tumor). *rtTA* (n=10), *rtTA/Plk4* (n=6), *rtTA/Raidd*$^{-/-}$ (n=4); *rtTA/Plk4/Raidd*$^{-/-}$ (n=4). Data is shown as mean ± SD. **(C)** Representative karyograms derived by mini-bulk sequencing of tumors (30 cells/tumor) isolated from mice of the indicated genotypes. **(D)** Representative western blots for p53 and p19ARF to estimate p53 function in respective tumors isolated from terminally ill mice with the indicated genotypes. Aneuploidy data were statistically tested by unpaired t test with Welch's correction. Centriole quantifications data were tested by Sidak's multiple comparisons test. *P < 0.05, **P < 0.01, ***P < 0.005, ns= not significant.
